# A Photo‐Patternable Solid‐State Electrolyte for High‐Performance, Miniaturized, and Implantable Organic Electrochemical Transistor‐Based Circuits

**DOI:** 10.1002/adma.202509314

**Published:** 2025-08-22

**Authors:** Miao Xiong, Chi‐Yuan Yang, Junpeng Ji, April S. Caravaca, Qi Guo, Qifan Li, Mary J. Donahue, Dace Gao, Han‐Yan Wu, Adam Marks, Yincai Xu, Deyu Tu, Iain McCulloch, Peder S. Olofsson, Simone Fabiano

**Affiliations:** ^1^ Laboratory of Organic Electronics Department of Science and Technology Linköping University Norrköping 60174 Sweden; ^2^ Laboratory of Immunobiology Center for Bioelectronic Medicine Department of Medicine, Solna Center for Molecular Medicine Karolinska Institutet Stockholm 17177 Sweden; ^3^ Wallenberg Wood Science Center Department of Science and Technology (ITN) Linköping University Norrköping 60174 Sweden; ^4^ Department of Chemistry University of Oxford Oxford OX1 3TA UK; ^5^ Andlinger Center for Energy and the Environment and Department of Electrical and Computer Engineering Princeton University Princeton, NJ 08544 USA; ^6^ Wallenberg Initiative Materials Science for Sustainability Department of Science and Technology Linköping University Norrköping 60174 Sweden

**Keywords:** implantable bioelectronics, integrated complementary logic circuits, organic electrochemical transistors, photo‐patternable solid‐state electrolyte

## Abstract

Organic electrochemical transistors (OECTs) are crucial for next‐generation (bio‐)electronic devices but are often constrained by the use of aqueous electrolytes, which introduce crosstalk, hinder miniaturization, and limit circuit integration. Here, a photo‐patternable solid‐state electrolyte based on 𝜄‐carrageenan (𝜄‐CGN) and poly(ethylene glycol) diacrylate (PEGDA) is presented, enabling high‐performance OECTs and complementary circuits. The 𝜄‐CGN electrolyte exhibits high ionic conductivity (>10 mS cm^−1^), comparable to a 0.1 m NaCl aqueous electrolyte, while supporting precise patterning down to 15 µm, fast transient response times, minimal hysteresis, and excellent stability in both p‐ and n‐type OECTs. Compact solid‐state NAND/NOR gates (500 × 800 µm^2^), 4‐input NAND gates (1600 × 800 µm^2^, 8 OECTs), and half‐adders (2 × 1 mm^2^, 18 OECTs) are demonstrated, all exhibiting correct logic functions and low‐voltage operation. To highlight its potential for implantable bioelectronics, solid‐state spiking circuits, monolithically integrated with flexible cuff electrodes, are developed for vagus nerve stimulation in mice. These findings establish 𝜄‐CGN‐based solid‐state electrolytes as a promising platform for scalable, implantable circuits, paving the way for next‐generation bioelectronic devices.

## Graphical abstract

A photo‐patternable solid‐state electrolyte based on 𝜄‐carrageenan (𝜄‐CGN) and poly(ethylene glycol) diacrylate (PEGDA) is developed to enable high‐performance complementary OECT circuits. The successful demonstration of fully implanted devices for vagus nerve stimulation in mice highlights solid‐state 𝜄‐CGN‐based electrolytes as a promising platform for scalable, implantable circuits and next‐generation bioelectronic technologies.

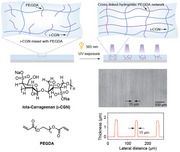

## 1 Introduction

Organic electrochemical transistors (OECTs) represent a rapidly advancing technology crucial for developing next‐generation (bio‐)electronic devices^[^ ^1^, ^2^, ^3^ ^]^. OECTs have been successfully used for various applications, including chemical/physical/biological sensors^[^ ^4^, ^5^ ^]^, tissue/brain activity monitoring devices^[^ ^6^, ^7^, ^8^, ^9^, ^10^ ^]^, active microelectrode arrays^[^ ^11^, ^12^ ^]^, nervetronics^[^ ^13^, ^14^ ^]^, neuromorphic hardware^[^ ^15^, ^16^, ^17^ ^]^, and neural interfaces^[^ ^18^, ^19^ ^]^. Despite their promise, a significant limitation of OECTs is their typical operation in aqueous liquid electrolytes. While these electrolytes facilitate high ion mobility, which is essential for bioelectronic applications, they also introduce challenges such as water leakage, which compromises device stability and hinders miniaturization. Moreover, the liquid nature of aqueous electrolytes can cause unwanted crosstalk between different devices on the same substrate, impeding their seamless integration into large‐area arrays. These issues become particularly critical for implantable OECT‐based circuits, where electrical isolation and long‐term stability are essential. Consequently, developing high‐performance OECTs based on solid‐state electrolytes is a pressing scientific challenge.

To date, two main categories of solid‐state electrolytes have been utilized in OECTs: polyelectrolytes and ion‐gel electrolytes. Polyelectrolytes, such as poly(sodium‐4‐styrene sulfonate) (PSSNa)^[^ ^20^, ^21^, ^22^ ^]^ and polyquaternium‐10 (PQ‐10)^[^ ^20^, ^23^ ^]^, feature ionizable groups along their backbone, which can dissociate in the presence of water, making the polymer capable of conducting ions. For example, Yu et al. developed a solid‐state electrolyte comprising a mixture of gelatin, PSSNa, D‐sorbitol, glycerin, NaCl, and deionized water, yielding OECTs with low threshold voltages (*V*_TH_ = 0.11 V) and good transconductances (*g*_m_ ≈ 2.14 mS) but much slower response time (τ ≈ 750 ms) compared to OECTs using NaCl aqueous electrolytes (≈0.3 ms)^[^ ^24^ ^]^. Ion‐gel electrolytes, consisting of a polymer matrix (e.g., polyvinyl alcohol (PVA)^[^ ^25^, ^26^, ^27^ ^]^, poly(vinylidene fluoride) (PVDF)^[^ ^27^, ^28^ ^]^, poly(ethylene oxide) (PEO)^[^ ^29^, ^30^ ^]^, poly(hydroxyethyl methacrylate) (PHEMA)^[^ ^31^ ^]^, or gelatin^[^ ^32^, ^33^, ^34^ ^]^), infused with ion liquids or salts, combine the flexibility and processability of polymers with the high ionic conductivity of ionic liquids. The polymer network provides structural support, while the ionic liquid or salt ensures conduction of ions through the gel. Although ion‐gel electrolytes like NaCl:PVA^[^ ^25^ ^]^, [EMIM][TFSI]:PVDF‐co‐HFP^[^ ^35^ ^]^, and [EMIM][EtSO_4_]:PNIPAm^[^ ^36^ ^]^ can yield good OECT performance, they exhibit noticeable hysteresis due to slow ion mobility. Recently, Tang et al. reported complementary circuits using a gelatin mixture with [MTEOA][MeOSO_3_], which showed good electrical performance and high stability^[^ ^34^ ^]^. However, the devices still exhibited overall slow off‐to‐on transient response times and high threshold voltages for p‐type OECTs and reduced *g*_m_ and charge carrier mobility × volumetric capacitance (µC*), as well as operational instability in the saturation regime for n‐type OECTs. Compared to liquid electrolytes (e.g., ≈10 mS cm^−1^ for 0.1 m NaCl)^[^ ^37^ ^]^, both polyelectrolytes and ion‐gel electrolytes exhibit significantly lower ionic conductivity (0.1–5 mS cm^−1^)^[^ ^38^, ^39^ ^]^ and higher RC time constants^[^ ^40^ ^]^. As a result, these materials lead to increased hysteresis and slower device response, typically <1 ms for OECTs operating with aqueous electrolytes^[^ ^41^, ^42^, ^43^, ^44^ ^]^, but exceeding 100 ms for those utilizing solid‐state electrolytes^[^ ^35^, ^45^, ^46^, ^47^ ^]^ (see Table S1, Supporting Information for a survey of the field).

Hydrogels, which are cross‐linked polymer networks infiltrated with water, offer exceptional advantages in bioelectronics due to their excellent biocompatibility, high water content that mimics ion‐rich physiological environments, and remarkable flexibility in design and fabrication ^[^ ^48^, ^49^, ^50^ ^]^. These properties make hydrogels a promising class of electrolytes for solid‐state OECTs. Despite their potential, only a limited number of studies have explored the use of hydrogels in solid‐state OECTs^[^ ^20^, ^25^, ^26^, ^31^, ^32^, ^51^ ^]^, and no reports to date have described the development of photo‐patternable water‐based hydrogels for OECT applications. A photo‐patternable, solid‐state electrolyte would enable the fabrication of independent gate electrodes, facilitating the miniaturization and integration of OECTs for implantation, where precise control over circuit architecture is crucial for bio‐signal monitoring and stimulation. Notably, implanted devices must be properly encapsulated to prevent crosstalk, a challenge that aqueous electrolytes cannot overcome. While there have been a few reports of implanted OECTs utilizing solid‐state electrolytes^[^ ^52^, ^53^ ^]^, most have been limited to single‐transistor architectures, and no studies have yet demonstrated fully integrated implantable OECT‐based circuits with smaller, more complex transistor networks.

Here, we report a photo‐patternable electrolyte for high‐performance solid‐state OECTs. Using 𝜄‐carrageenan (𝜄‐CGN) as the ion conductor and poly(ethylene glycol) diacrylate (PEGDA) as the crosslinker, we developed a solid‐state electrolyte that exhibits ionic conductivity exceeding 10 mS cm^−1^, comparable to that of 0.1 m NaCl aqueous electrolytes. The 𝜄‐CGN‐based electrolyte enables precise patterning down to 15 µm, fast transient response times (*τ*_ON_ = 0.66 ms and *τ*_OFF_ = 0.18 ms for n‐type OECTs, *τ*_ON_ = 0.21 ms and *τ*_OFF_ = 0.038 ms for p‐type OECTs), and excellent operational stability. Complementary inverters based on these solid‐state OECTs exhibit gains exceeding 70 VV^−1^ and power consumption of less than 2 µW. Furthermore, we demonstrated solid‐state complementary OECT‐based NAND and NOR gates with footprints of 500 × 800 µm^2^, as well as half‐adders composed of 18 OECTs occupying an area of just 2 mm^2^. The half‐adder exhibited excellent logic output with fast operation speed and low operating voltage down to 0.6 V. Additionally, to demonstrate the potential of 𝜄‐CGN‐based solid‐state electrolytes for enabling highly integrated bioelectronic circuits, we developed solid‐state organic electrochemical neurons (OECNs) capable of generating voltage spikes with amplitudes of 0.3–0.4 V and frequencies of 1–20 Hz. When encapsulated with a biocompatible parylene layer and monolithically integrated with flexible cuff electrodes on a conformable substrate, these solid‐state spiking circuits can be fully implanted to enable vagus nerve stimulation in rodents, highlighting their promise for applications in implantable bioelectronics.

## 2 Results and Discussion

### 2.1 Design and Properties of Photo‐Patternable 𝜄‐CGN‐Based Electrolytes

**Figure 1:**
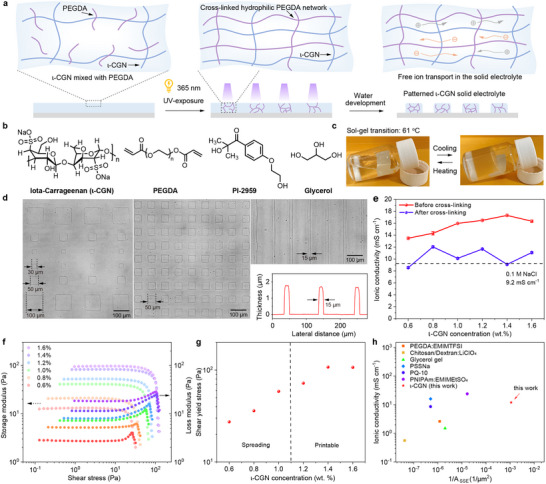
a) Schematic of the crosslinking process of 𝜄‐CGN via UV exposure. Under 365 nm UV light, PEGDA crosslinks to form a solid electrolyte. Unexposed regions are removed by water development, yielding patterned areas where ions can freely move. b) Chemical structures of 𝜄‐CGN, PEGDA, photoinitiator PI‐2959, and glycerol. c) Solution‐gel transition of 𝜄‐CGN‐based electrolyte upon cooling/heating. d) Optical microscope images of cross‐linked 𝜄‐CGN electrolyte patterned into 100 × 100, 50 × 50, 30 × 30 µm^2^ pixel arrays. Optical image and height profile of a 15‐µm‐wide line array. e) Ionic conductivity of 𝜄‐CGN‐based electrolyte before and after cross‐linking at various 𝜄‐CGN concentrations. Error bars represent standard deviation (*n* = 3). f) Storage and loss moduli as a function of shear stress for uncrosslinked 𝜄‐CGN electrolyte at different concentrations. g) Shear yield stress of the 𝜄‐CGN electrolyte as a function of 𝜄‐CGN concentration, extracted from rheological tests. h) Ionic conductivity vs. the smallest patterned solid‐state electrolyte area (A_SSE_) reported in the literature.

To develop solid‐state OECTs, we designed a photo‐patternable solid‐state electrolyte using sequential ultraviolet (UV) light‐triggered solubility modulation^[^ ^54^ ^]^ (**Figure** **1**a). 𝜄‐CGN (Figure 1b), a natural sulfated polysaccharide extracted from edible red seaweeds and widely used as a stabilizing, thickening, and gelling agent in food, cosmetic, and pharmaceutical industries^[^ ^55^ ^]^, was selected as the ion conductor. Due to its stability, biocompatibility, and non‐toxic nature, 𝜄‐CGN is extensively employed in drug delivery systems, encapsulation technologies, and biomedical applications, making it particularly suitable for bioelectronic devices. Structurally, 𝜄‐CGN features a charged polymer backbone with Na⁺ and sulfate ions, enabling higher ionic conductivity than many conventional solid‐state electrolytes. UV‐sensitive PEGDA and 2‐hydroxy‐4'‐(2‐hydroxyethoxy)‐2‐methylpropiophenone (PI‐2959) were used as the crosslinker and photoinitiator, respectively (Figure 1b). Upon UV light irradiation (𝜆 = 365 nm, 40 mW cm^−2^ for 15 s), PEGDA covalently crosslinks in the presence of PI‐2959, forming a hydrogel network with 𝜄‐CGN that is water‐resistant. The unexposed regions remain soluble and can be washed away with water, enabling precise patterning of the 𝜄‐CGN electrolyte (Figure 1a). The cross‐linked 𝜄‐CGN electrolyte appears as a white, soft solid, which can be squeezed and stretched (Figure S1, Supporting Information). Using direct optical lithography (390 nm, 5000 mJ cm^−2^), the 𝜄‐CGN‐based electrolyte can be photopatterned into uniformly distributed small pixels with lateral dimensions as small as 15 µm (Figure 1d). This capability highlights its potential for integrating miniaturized solid‐state OECT‐based complementary circuits into large‐area arrays.

We then characterized the ionic conductivity of the solid‐state 𝜄‐CGN‐based electrolyte by sandwiching it between two gold electrodes and measuring impedance across different frequencies (100 Hz–100 MHz) and 𝜄‐CGN concentrations (0.6–1.6 wt.%) using electrochemical impedance spectroscopy (EIS) (Figure 1e and Figures S2, S3, Supporting Information). The resulting Nyquist plot data were fitted using an equivalent circuit with a resistance and a constant phase element in series (Figures S4, S5, Supporting Information). The ionic conductivity of 𝜄‐CGN electrolyte increases with higher concentrations of 𝜄‐CGN, reaching over 17 mS cm^−1^ at a concentration of 1.4 wt.% (Figure 1e). After UV cross‐linking, the ionic conductivity reduces slightly but remains above 10.0 mS cm^−1^, on par with the measured ion conductivity of a 0.1 m NaCl aqueous electrolyte (9.2 mS cm^−1^), holding potential for the development of fast solid‐state OECTs. We attribute the drop in ionic conductivity after cross‐linking to changes in the hydrogel's network density, which can restrict ion diffusion^[^ ^56^ ^]^. Despite this minor reduction in conductivity, the values remain well within the range required for efficient OECT operation, ensuring minimal impact on device performance. Compared to previously reported patternable electrolytes, the ι‐CGN‐based electrolyte exhibits higher ion conductivity, a lower sol‐gel transition temperature (see below), and water solubility before cross‐linking, while becoming water‐insoluble afterward, enabling high‐resolution patterning via direct photolithography (Figure 1h and Table S2, Supporting Information).

To investigate the electrolyte's rheological properties, we measured storage modulus (G′) and loss modulus (G″) under varying shear stress (Figure 1f). The 𝜄‐CGN electrolyte exhibits characteristic shear‐thinning and shear‐yielding behavior. In the linear viscoelastic region (G′ > G″), the electrolyte is in a gel state, but as the shear stress increases, G’ and G″ intersect, marking the transition to flow dynamics (G′ < G″). This intersection point, known as the flow point, serves as a viscosity benchmark (Figure 1g). Electrolytes with lower 𝜄‐CGN concentrations (0.6–1.0 wt.%) exhibit lower viscosity and yield stress, leading to lateral spreading on substrates (Figure S6, Supporting Information) and making them suitable for spin coating. In contrast, higher concentrations (1.2–1.6 wt.%) result in increased viscosity and yield stress (Figure S6, Supporting Information), ideal for applications like 3D printing (Figure S7, Supporting Information). Additionally, analysis of 𝜄‐CGN rheological properties as a function of temperature revealed a low sol‐gel transition temperature of 61 °C, with the hydrogel transitioning into a solution upon heating to 100 °C and reverting to a gel upon cooling (Figure 1c and Figure S8, Supporting Information).

### 2.2 Electrical Characteristics of Solid‐State p‐ and n‐type OECTs

**Figure 2:**
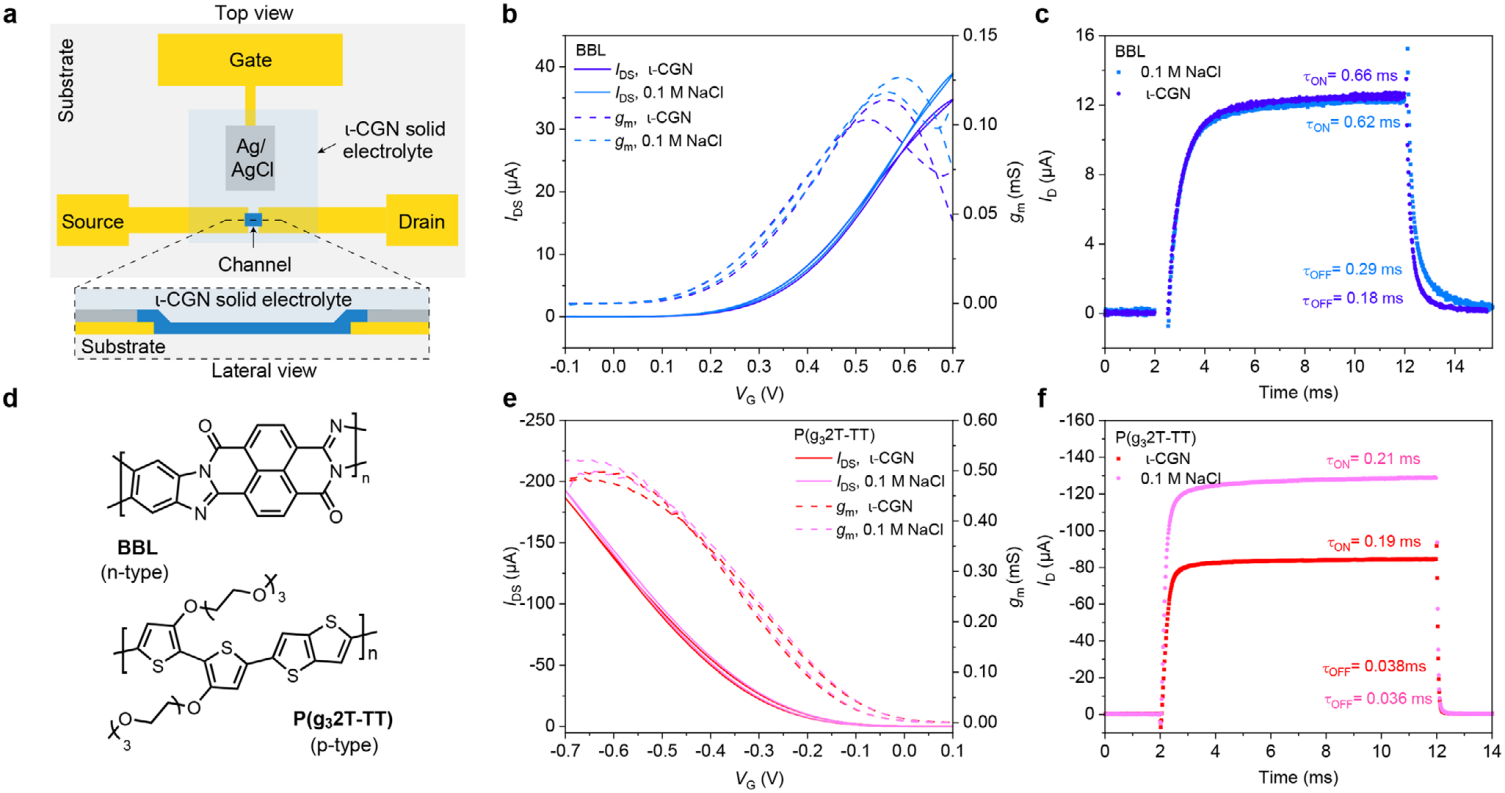
a) Schematic of a solid‐state OECT based on 𝜄‐CGN electrolyte. b) Transfer characteristics and transconductance of BBL‐based OECTs with solid‐state 𝜄‐CGN electrolyte and liquid 0.1 m NaCl electrolyte measured at *V*_DS_ = −0.7 V (channel dimensions: *W* = 100 µm, *L* = 10 µm, and *d* = 20 nm). c) Transient response of BBL‐based OECTs with solid‐state 𝜄‐CGN electrolyte and liquid 0.1 m NaCl electrolyte (same dimensions as in b). d) Chemical structures of the n‐type polymer BBL and p‐type polymer P(g_3_2T‐TT). e) Transfer characteristics and transconductance of P(g_3_2T‐TT)‐based OECTs with solid‐state 𝜄‐CGN electrolyte and liquid 0.1 m NaCl electrolyte measured at *V*_DS_ = −0.7 V (channel dimensions: *W* = 100 µm, *L* = 10 µm, and *d* = 5 nm). f) Transient response of P(g_3_2T‐TT)‐based OECTs with solid‐state 𝜄‐CGN electrolyte and liquid 0.1 M NaCl electrolyte (same dimensions as in e).

We then assessed the performance of the 𝜄‐CGN electrolyte in both p‐ and n‐type OECTs, using the hole‐transporting oligo(ethylene glycol)‐functionalized polythiophene P(g_3_2T‐TT)^[^ ^42^ ^]^ and the electron‐transporting poly(benzimidazobenzophenanthroline) (BBL)^[^ ^57^ ^]^ as prototypical channel materials (**Figure** **2**a,d). OECTs were prepared following the procedure described in the Experimental Section. Briefly, gold source and drain electrodes were deposited on a glass substrate and coated with a 4 µm‐thick Parylene C (PaC) layer. P(g_3_2T‐TT) and BBL thin films were cast and patterned using an additional PaC layer to define the OECT channel length (*L* = 10 µm) and width (*W* = 100 µm). The 𝜄‐CGN electrolyte was heated at 100 °C to form a solution, spin‐coated onto the channel, and patterned via UV light (365 nm) followed by water development. For comparison, we also fabricated similar OECTs using 0.1 m NaCl as the aqueous electrolyte. The results are summarized in Table S3 (Supporting Information).

Solid‐state BBL‐based OECTs exhibit typical n‐type accumulation‐mode output and transfer characteristics, achieving ON/OFF current ratios exceeding 10^5^, comparable to BBL‐based OECTs with NaCl aqueous electrolytes (Figure 2b,c, and Figure S9f, Supporting Information). The devices demonstrate excellent reproducibility across different 𝜄‐CGN concentrations (Figures S9–S12, Supporting Information). No hysteresis was observed over the entire *V*_G_ range, a key requirement for stable analog circuit operation. A maximum *g*_m_ of 0.11 mS was recorded, similar to the 0.12 mS measured for devices with NaCl liquid electrolyte (Figure 2b). The solid‐state BBL‐based OECTs exhibit an average geometry‐normalized transconductance (*g*_m,norm_) of 6.0 ± 0.84 S cm^−1^, a µC* product of 21.34 ± 1.52 F cm^−1^ V^−1^ s^−1^, and a *V*_th_ of 0.17 ± 0.01 V. These values align closely with those of BBL OECTs using NaCl aqueous electrolytes (*g*_m,norm_ = 5.50 ± 1.02 S cm^−1^, µC* = 21.15 ± 1.48 F cm^−1^ V^−1^ s^−1^ and *V*_th_ = 0.17 ± 0.02 V, Table S3, Supporting Information)^[^ ^57^ ^]^. Note that the drop in drain current (*I*_D_) observed in the linear regime (*V*_D_ < 0.2 V) at *V*_G_ > 0.6 V (Figure S9, Supporting Information) results from the characteristic antiambipolar behavior of BBL^[^ ^7^, ^58^ ^]^.

The switching speed of solid‐state BBL‐based OECTs was evaluated by pulsing *V*_G_ between 0 and 0.6 V while recording *I*_D_ over time. Rise (*τ*_ON_) and decay (*τ*_OFF_) times were extracted using exponential fits (Figure 2c, see also Figure S11 and Table S4, Supporting Information). Response times were also estimated by measuring the time for *I*_D_ to reach 90% of its max (*τ*_ON,90%_) and min (*τ*_OFF,90%_) values under the same *V*_G_ pulsing conditions (Table S4, Supporting Information). At 𝜄‐CGN concentrations below 1.2 wt.%, the transient response time was 0.84 ± 0.04 ms (*τ*_ON,90%_ = 3.06 ± 0.24 ms), improving to *τ*_ON_ = 0.66 ± 0.02 ms (*τ*_ON,90%_ = 2.28 ± 0.18 ms) and *τ*_OFF_ = 0.18 ± 0.01 ms (*τ*_OFF,90%_ = 0.49 ± 0.04 ms) at 1.6 wt.%. These values are comparable to those of BBL OECTs with NaCl solution (*τ*_ON_ = 0.62 ± 0.03 ms and *τ*_OFF_ = 0.29 ± 0.02 ms) (Figure S13d, Supporting Information). In addition, BBL‐based OECTs with 𝜄‐CGN electrolyte exhibit exceptional stability (Figure S13e, Supporting Information), retaining over 98% of their initial current after more than 600 switching cycles and over 1 h of continuous operation post‐encapsulation (see Experimental Section for details on the encapsulation approach).

Solid‐state P(g_3_2T‐TT) OECTs also demonstrate excellent performance, comparable to those using NaCl aqueous electrolyte (Figure 2e,f, see also Figures S13, S14 and Table S3, Supporting Information). Devices with the 𝜄‐CGN solid‐state electrolyte achieve an average µC* of 68.06 F cm^−1^ V^−1^ s^−1^ and *g*_m,norm_ of 24.5 S cm^−1^, while those with NaCl solution reach 74.29 F cm^−1^ V^−1^ s^−1^ and *g*_m,norm_ of 26 S cm^−1^, respectively. The P(g_3_2T‐TT) OECTs with solid‐state 𝜄‐CGN electrolyte exhibit fast transient response times (*τ*_ON_ = 0.21 ± 0.01 ms and *τ*_OFF_ = 0.038 ± 0.002 ms), closely matching those with NaCl solution (*τ*_ON_ = 0.19 ± 0.02 ms and *τ*_OFF_ = 0.036 ± 0.002 ms) (Figure 2f). Additionally, solid‐state P(g_3_2T‐TT)‐based OECTs exhibit significantly higher stability, retaining ≈50% of their initial current after over 1 h of continuous operation (Figure S13f, Supporting Information). In contrast, devices with NaCl aqueous electrolytes retain only 10% of their initial current after prolonged use, consistent with previous reports^[^ ^59^ ^]^. The instability of P(g_3_2T‐TT) is well known and common to other low ionization energy polythiophene‐based polymers, where oxidative side reactions contribute to current loss over time^[^ ^60^ ^]^. Nonetheless, these results indicate that the 𝜄‐CGN‐based electrolyte enables the development of solid‐state p‐ and n‐type OECTs without compromising device performance.

### 2.3 Solid‐State Complementary Circuits Based on 𝜄‐CGN Electrolyte

**Figure 3:**
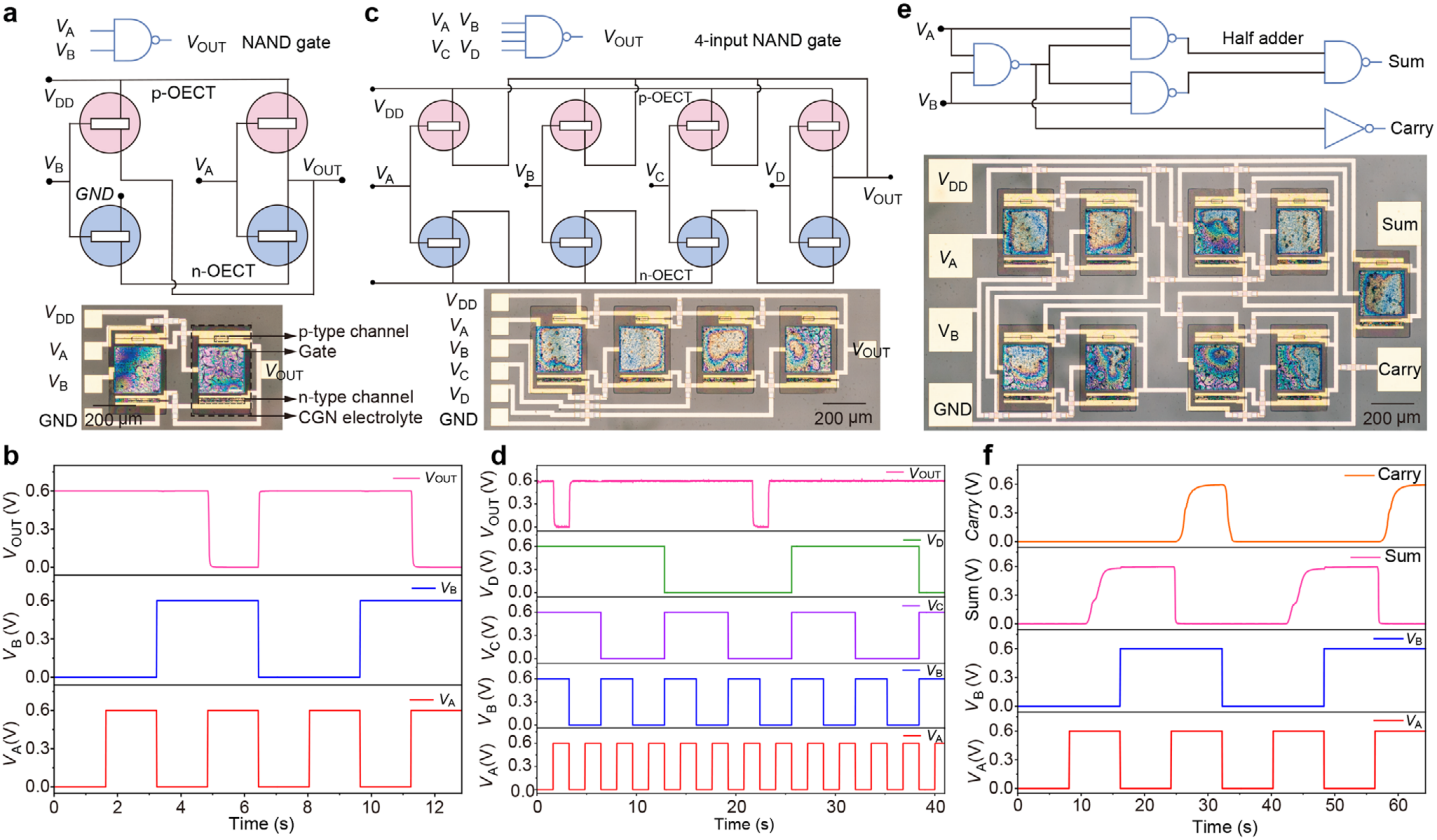
a) Schematic and photograph of a NAND gate (500 × 800 µm^2^) based on 4 OECTs: two p‐type P(g_3_2T‐TT)‐based OECTs and two n‐type BBL‐based OECTs with patterned solid‐state 𝜄‐CGN electrolyte. The gate is shared by the p‐ and n‐type channels. b) Voltage output characteristics of the NAND gate. c) Schematic and photograph of a 4‐input NAND gate (1600 × 800 µm^2^) based on 8 OECTs. d) Voltage output characteristics of the 4‐input NAND gate. e) Schematic and photograph of a half‐adder circuit (2 × 1 mm^2^) comprising 18 OECTs. f) Voltage output characteristics of the half‐adder. The P(g_3_2T‐TT)‐OECTs have dimensions: *W* = 50 µm, *L* = 4 µm, and *d* = 5 nm. The BBL‐OECTs have dimensions: *W* = 200 µm, *L* = 4 µm, and *d* = 20 nm. Truth tables of the circuits are provided in Table S7 (Supporting Information).

Having demonstrated the excellent performance of the 𝜄‐CGN electrolyte, we proceeded to fabricate complementary circuits using solid‐state P(g_3_2T‐TT) and BBL OECTs (**Figure** **3**a–e, Figures S15–S20, Supporting Information). Detailed fabrication steps are provided in the Experimental Section and Figure S15, Supporting Information. The p‐type P(g_3_2T‐TT) OECTs were designed with a channel geometry of *W*/*L* = 50/4 µm µm^−1^, while the n‐type BBL OECTs used *W*/*L* = 200/4 µm µm^−1^. The polymeric channels were patterned using a sacrificial PaC layer, with thicknesses of 5 nm (P(g_3_2T‐TT)) and 20 nm (BBL) optimized for well‐balanced p‐/n‐channel electrical characteristics (Figure S16, Supporting Information), ensuring comparable current levels even in the sub‐threshold region at *V*_G_ ≈|0.1| V. The 𝜄‐CGN electrolyte was spin‐coated and patterned (250 × 250 µm^2^) via direct laser writing, with a shared gate electrode to minimize device size. The resulting solid‐state complementary inverters, with a footprint of 250 × 400 µm^2^, are among the smallest reported for planar OECT‐based inverters (Figure 3a and Table S5, Supporting Information)^[^ ^61^ ^]^. The balanced p‐/n‐type OECTs enable operation at supply voltages (*V*_DD_) as low as 0.1 V, achieving a switching threshold (*V*_M_) of 0.071 V, a voltage gain of 2.1 VV^−1^, static power consumption of 0.22 nW, and peak power consumption during switching of 1.7 nW. A maximum gain of 70 VV^−1^ was recorded at *V*_DD_ = 0.7 V, with peak power consumption remaining below 2 µW (Figure S17, Supporting Information). These performance metrics closely match those of NaCl aqueous electrolyte (Figure S18, Supporting Information) and rank among the highest reported for OECT‐based technologies^[^ ^20^, ^23^, ^34^, ^57^, ^62^, ^63^ ^]^.

After demonstrating high‐performance solid‐state complementary OECT‐based inverters, we fabricated compact solid‐state complementary NAND, NOR, 4‐input NAND gates, and half‐adders, key functional units for digital integrated circuits used in signal processing and computing. The smallest NAND and NOR gates (500 × 800 µm^2^), consisting of 4 OECTs (two p‐type and two n‐type) with solid‐state 𝜄‐CGN electrolyte pixels, were fabricated via direct optical lithography (Figure 3a and Figure S19, Supporting Information). By varying the input voltage from 0 to 0.6 V, the output voltage switched between 0.6 V and ground, with both gates operating as expected, producing correct logic outputs (1 or 0) based on input states (0 or 1) at voltage bias of only 0.6 V (Figure 3b and Figure S19b, Supporting Information). The NAND gates achieved an operational speed of 0.4 s (Figure S20, Supporting Information), ranking among the fastest reported for OECTs‐based NAND gates (Table S6, Supporting Information)^[^ ^61^, ^62^, ^64^ ^]^. A 4‐input NAND gate (1600 × 800 µm^2^), built from 8 OECTs, correctly produced an output of 0 when all four inputs were high (1) (Figure 3c), demonstrating the feasibility of combinational logic implementation with multiple inputs (Figure 3d). Additionally, we developed a more complex logic circuit, a half adder composed of 18 OECTs with a footprint of just 2 × 1 mm^2^, delivering rail‐to‐rail outputs with fast operation (Figure 3e,f). These results highlight the seamless integration and functionality of solid‐state OECTs, which is unattainable with aqueous electrolytes at these scales. Notably, this half‐adder is fully based on OECTs and demonstrates an unprecedented level of integration, incorporating one of the largest numbers of OECTs reported in a complementary OECT‐based circuit (Table S5, Supporting Information).

### 2.4 Implanted Solid‐State OECT‐Based Spiking Circuits for Nerve Stimulation

**Figure 4:**
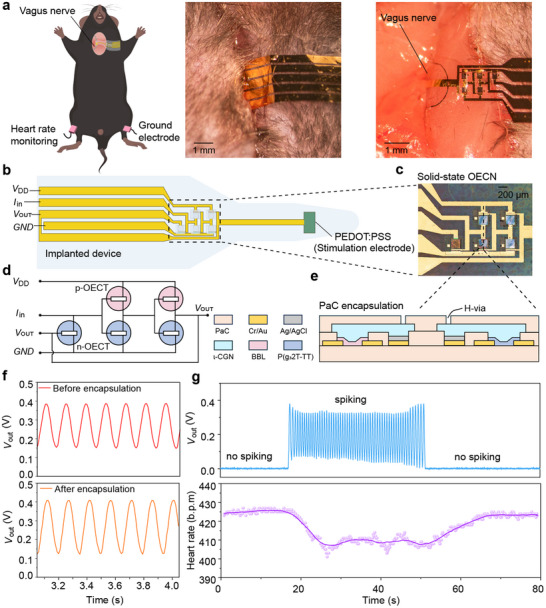
a) Schematic and photograph of the implanted, solid‐state OECN‐based stimulator in a mouse, with the stimulation electrode wrapped around the vagus nerve. b) Schematic of the implanted device. c) Photograph of the solid‐state OECN. d) Circuit diagram of the solid‐state OECN. e) Lateral‐view schematic of the solid‐state OECN, showing PaC encapsulation for protection. f) Spiking behavior of the solid‐state OECN before and after encapsulation, recorded at *I*_in_ = 1 µA and *V*_DD_ = 0.6 V. The OECT geometry is as follows: P(g_3_2T‐TT)‐OECTs with *W* = 200 µm, *L* = 4 µm, and *d* = 5 nm, and BBL‐OECTs with *W* = 800 µm, *L* = 4 µm, and *d* = 20 nm. g) Example of the output response of the implanted, solid‐state OECN (same device as in f) and the corresponding heart rate reduction during stimulation. Note: The observed drop in OECN spiking frequency after connection to the mouse arises from the mouse acting as an external load, which dampens the frequency.

To demonstrate the potential of 𝜄‐CGN electrolytes for bioelectronic applications, we developed solid‐state organic electrochemical neurons (OECNs) monolithically integrated with flexible cuff electrodes on a PaC substrate for vagus nerve stimulation (**Figure** **4**a–e). OECNs are bioinspired spiking circuits that convert ionic and electronic signals into action‐potential‐like spikes, enabling seamless interfacing with biological systems and making them highly relevant for neuromorphic bioelectronics and neurostimulation applications^[^ ^15^ ^]^. These artificial neurons process and transmit information through transient electrical spikes, thereby mimicking the functioning of the nervous system. However, previously reported OECNs relied on liquid electrolytes, which limit miniaturization, stability, and suitability for implantation. The use of a solid‐state 𝜄‐CGN electrolyte can overcome these challenges and enable compact, stable, and fully integrated OECNs, making practical bioelectronic interfaces possible.

The OECN used here is a bio‐inspired event‐based sensor based on a leaky integrate‐and‐fire (LIF) Axon‐Hillock model^[^ ^13^ ^]^, implemented with a solid‐state 𝜄‐CGN electrolyte (Figure 4b–e). The circuit consists of an amplifying block, built from complementary n‐type (BBL) and p‐type (P(g_3_2T‐TT)) OECTs, and a reset transistor (BBL‐OECT), all fabricated on a 2.5‐µm‐thin PaC substrate using the same fabrication process as the logic circuits. Spikes are generated by integrating the input current (*I*_in_) at the input terminal. To ensure stability and hydration during implantation, the devices were encapsulated with an additional 1.5 µm PaC layer, and a vertical hydration via (H‐via)^[^ ^53^ ^]^ was added to prevent crosstalk between the different parts of the LIF‐OECN through body fluids (Figure 4e). Encapsulation did not alter the firing frequency, which remained stable at ≈7 Hz before and after encapsulation (Figure 4f). Reducing the channel area increased the spiking frequency to ≈20 Hz (Figure S21, Supporting Information). The spiking frequency can also be tuned by adjusting *I*_in_^[^ ^13^ ^]^, enabling programmable stimulation suitable for implantable neurostimulation applications.

The solid‐state OECN was then monolithically integrated with a flexible gold stimulation electrode coated with poly(3,4‐ethylenedioxythiophene):polystyrene sulfonate (PEDOT:PSS) to reduce impedance^[^ ^65^ ^]^. The OECN and PEDOT:PSS‐based electrodes were then wrapped around the right cervical vagus nerve of a mouse, and the heart rate was monitored over time (Figure 4a). The “wrap‐around” method avoids penetrating the protective nerve sheath, minimizing damage, and is standard in clinical vagus nerve stimulation (VNS) applications.^[^ ^66^ ^]^ When the OECN was off (no spiking), the mouse's heart rate remained at a stable baseline, indicating good post‐implantation condition without adverse effects. When activated (spiking), the OECN reduced heart rate by ≈2–4% (Figure 4g), a reduction level consistent with VNS at ≤ 5 Hz^[^ ^67^ ^]^. This result confirms that solid‐state OECNs can be implanted and effectively stimulate nerves, overcoming the limitations of liquid‐electrolyte‐based devices. Since VNS is already used clinically for treating depression^[^ ^68^ ^]^, epilepsy^[^ ^69^ ^]^, and inflammatory diseases^[^ ^70^ ^]^, this demonstration highlights the potential of fully implanted, solid‐state OECN‐based systems for closed‐loop physiological regulation.

## 3 Conclusion

In conclusion, we have developed a photo‐patternable solid‐state 𝜄‐CGN electrolyte for high‐performance OECTs and OECT‐based complementary circuits. The 𝜄‐CGN electrolyte exhibits high ionic conductivity (>10 mS cm^−1^), comparable to 0.1 m NaCl liquid electrolytes, and enables precise patterning down to 15 µm. This facilitates fast electrochemical response, minimal hysteresis, high OECT performance, and excellent operational stability in both p‐ and n‐type devices. Solid‐state complementary inverters achieved gains up to 70 VV^−1^ with low power consumption (<2 µW). We fabricated compact (500 × 800 µm^2^) solid‐state NAND/NOR gates and 4‐input NAND gates (1600 × 800 µm^2^, 8 OECTs), all demonstrating correct logic functions. We also developed half‐adders (2 × 1 mm^2^) integrating 18 OECTs, which operated at low voltages (down to 0.6 V) and exhibited excellent logic output. Beyond logic circuits, we demonstrated solid‐state spiking circuits monolithically integrated with flexible cuff electrodes for vagus nerve stimulation in mice. Together, these results establish the 𝜄‐CGN solid‐state electrolyte as a viable platform for stable, miniaturized, and precise neurostimulation in implantable bioelectronics, paving the way for next‐generation bioelectronic therapies.

## 4 Experimental Section

### Materials

PEGDA, ι‐carrageenan, and PI‐2959 were purchased from Sigma–Aldrich. BBL and P(g_3_2T‐TT) were synthesized following previous literature^[^ ^57^ ^]^.

### Sample Preparation

P(g_3_2T‐TT) (3 mg) was dissolved in dichloroethane (1 mL) to form a dark blue solution (3 mg mL^−1^). BBL (3 mg) was dissolved in methanesulfonic acid (MSA) (1.5 mL) to form a deep red solution (2 mg mL^−1^). For the electrolyte, 1.5 g of PEGDA (700 Da), 200 mg of PI‐2959, 60–160 mg (0.6%–1.6%) of 𝜄‐CGN, and 10 g of H_2_O were added to a 20 mL bottle. NaCl was added to match the ionic concentration of a 0.1 m NaCl aqueous electrolyte. Glycerol was added to enhance the ionic conductivity, plasticity, water retention, and processability of the electrolyte. The mixture was heated to 100 °C for 30 min to form a uniform, transparent solution with high fluidity. The 𝜄‐CGN electrolyte was cooled down to room temperature to form a non‐flowing transparent gel.

### Fabrication and Characterization of OECTs, Inverters, NAND, NOR, and Half Adder

OECTs were fabricated following a previously reported procedure.^[^ ^71^ ^]^ Glass wafers were cleaned with acetone, deionized water, and isopropyl alcohol, and then dried with nitrogen. Source and drain electrodes (5 nm Cr and 50 nm Au) were thermally deposited and patterned photolithographically by wet etching. A 1 µm layer of Parylene C, along with a small amount of 3‐(trimethoxysilyl) propyl methacrylate (A‐174 Silane) to enhance adhesion, was deposited to act as an insulating layer, preventing capacitive effects at the metal‐liquid interface. Subsequently, a 2% Decan‐90 industrial surfactant solution was spin‐coated as an antiadhesive layer, and a 2 µm layer of Parylene C was deposited as a sacrificial layer. To protect the Parylene C layers from plasma reactive ion etching, a 5 µm thick positive photoresist (AZ10XT520CP) was spin‐coated on the Parylene C layer. Photolithographic patterning was then used to define the contact pads and the OECT channel, followed by the application of AZ developer to the photoresist. Plasma reactive ion etching was used to remove organic materials, including both the photoresist and Parylene C, leaving the electrode pads and channels patterned, while other areas remained covered by the two layers of Parylene C. A 100‐nm‐thick Ag layer was evaporated onto the Parylene C layer, then converted to Ag/AgCl by dipping it into a 1 mm HCl aqueous solution for 1 min. The Ag/AgCl gate was further patterned by lifting off the sacrificial parylene layer. For n‐type channel patterning, an antiadhesive layer and a 2‐µm‐thick Parylene C were first deposited as sacrificial layers. The channel region was defined by photolithography and etching. The BBL‐MSA solution was spin‐coated onto the Parylene C layer, followed by immersion in water and drying under nitrogen. The BBL was then patterned by peeling off the sacrificial Parylene C layer. The process for p‐type channel patterning was similar to that for the n‐type channel. After deposition of the antiadhesive and sacrificial Parylene C layers, the p‐type channel region was defined by photolithography and etching. After removing the sacrificial parylene, a 5‐nm‐thick P(g_3_2T‐TT) was deposited. For the OECTs with liquid electrolyte, a PDMS well was filled with a 0.1 m NaCl aqueous solution. For the solid‐state 𝜄‐CGN electrolyte, 1.5 g of PEGDA, 0.2 g of PI‐2959, 0.18 g of 𝜄‐CGN, and 0.3 g of glycerin were added to 10 g of water, then heated and stirred at 110 °C for 30 min. The hot electrolyte solution was spin‐coated at 1500 rpm for 15 s to form a 1‐µm‐thick transparent film, which was crosslinked by photolithography using a 390 nm UV light (5000 mJ cm^−^^2^). The device was then developed in water, immersed in 0.1 m NaCl in glycerin for 30 min, and gently dried with nitrogen flow. To encapsulate the OECT devices, paraffin wax was melted at 55 °C and quickly applied to cover the devices. Extended electrodes were left exposed for testing. The n‐type OECTs had a channel geometry of W/L = 100 µm/10 µm with a thickness of 20 nm. The p‐type OECTs had a channel geometry of W/L = 100 µm/10 µm with a thickness of 5 nm. For the NAND, NOR, and half‐adder, the p‐type OECTs had a channel of *W* = 50 µm, *L* = 4 µm, and *d* = 5 nm, while the n‐type OECTs had a channel of *W* = 200 µm, *L* = 4 µm, and *d* = 20 nm. The devices were characterized by using a Keithley 4200A‐SCS and a Keithley 2400 as an additional power supply.

### Modulation of the Biological Signal by Implanted Solid‐State OECN

C57BL/6J male mice (10–12 weeks old) were purchased from Charles River Laboratories and housed under a 12 h light/dark cycle with ad libitum access to food and water. The experimental protocol used was approved by the Stockholm Animal Research Ethics Committee (permits 20818‐2020 and 5466‐2025). The surgical procedure and vagus nerve isolation followed a previously described method^[^ ^72^ ^]^. Briefly, mice were anesthetized with isoflurane and an equal mixture of oxygen and air. Mice were placed on a heating pad in the supine position. An incision to the midline cervical area was made to expose the salivary glands and subcutaneous tissue. After separation of the tissue, the cervical vagus nerve and carotid artery were exposed. The vagus nerve was then isolated and immobilized with a suture to facilitate placement of the electrode.

## Conflict of Interest

The authors declare no conflict of interest.

## Supporting information



Supporting Information

## Data Availability

The data that support the findings of this study are available from the corresponding author upon reasonable request.
