# Supplementary material for: A Photo‐Patternable Solid‐State Electrolyte for High‐Performance, Miniaturized, and Implantable Organic Electrochemical Transistor‐Based Circuits
Source: Adv Mater. 2025 Aug 22;37(44):e09314. doi: 10.1002/adma.202509314 (PMC12592903; doi:10.1002/adma.202509314)
Supplement: Supplementary file 1 — Supporting Information [file ADMA-37-e09314-s001.pdf]

ADVANCED MATERIALS

Supporting Information

for *Adv. Mater.*, DOI 10.1002/adma.202509314

A Photo-Patternable Solid-State Electrolyte for High-Performance, Miniaturized, and Implantable Organic Electrochemical Transistor-Based Circuits

Miao Xiong, Chi-Yuan Yang, Junpeng Ji, April S. Caravaca, Qi Guo, Qifan Li, Mary J. Donahue, Dace Gao, Han-Yan Wu, Adam Marks, Yincai Xu, Deyu Tu, Iain McCulloch, Peder S. Olofsson and Simone Fabiano**

Supporting Information

A photo-patternable solid-state electrolyte for high-performance, miniaturized, and implantable organic electrochemical transistor-based circuits

Miao Xiong, Chi-Yuan Yang, Junpeng Ji, April S. Caravaca, Qi Guo, Qifan Li, Mary J. Donahue, Dace Gao, Han-Yan Wu, Adam Marks, Yincai Xu, Deyu Tu, Iain McCulloch, Peder S. Olofsson, Simone Fabiano**

Miao Xiong, Chi-Yuan Yang, Junpeng Ji, Qifan Li, Mary J. Donahue, Dace Gao, Han-Yan Wu, Yincai Xu, Deyu Tu, Simone Fabiano
Laboratory of Organic Electronics, Department of Science and Technology, Linköping University, 60174 Norrköping, Sweden.
E-mail: chi-yuan.yang@liu.se, simone.fabiano@liu.se

April S. Caravaca, Qi Guo, Peder S. Olofsson
Laboratory of Immunobiology, Center for Bioelectronic Medicine, Department of Medicine, Solna, Center for Molecular Medicine, Karolinska Institutet, 17177 Stockholm, Sweden.

Adam Marks, Iain McCulloch
Department of Chemistry, University of Oxford, OX1 3TA Oxford, UK.

Iain McCulloch
Andlinger Center for Energy and the Environment and Department of Electrical and Computer Engineering, Princeton University, Princeton, NJ, 08544, USA.

Mary J. Donahue, Simone Fabiano
Wallenberg Wood Science Center, Department of Science and Technology (ITN), Linköping University, 60174 Norrköping, Sweden.

Simone Fabiano
Wallenberg Initiative Materials Science for Sustainability, Department of Science and Technology, Linköping University, 60174 Norrköping, Sweden.

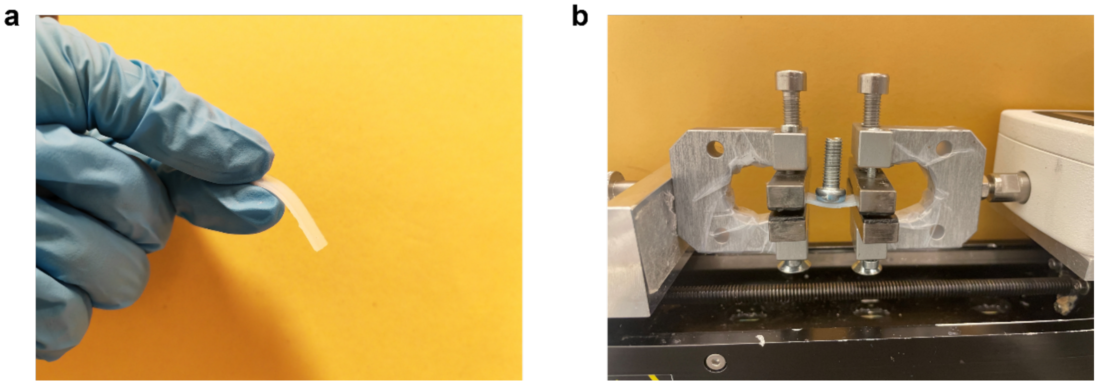

Figure S1. a) Free-standing crosslinked ι-CGN electrolyte. b) Crosslinked ι-CGN electrolyte pressed by heavy objects. Film thickness: 500 μm.

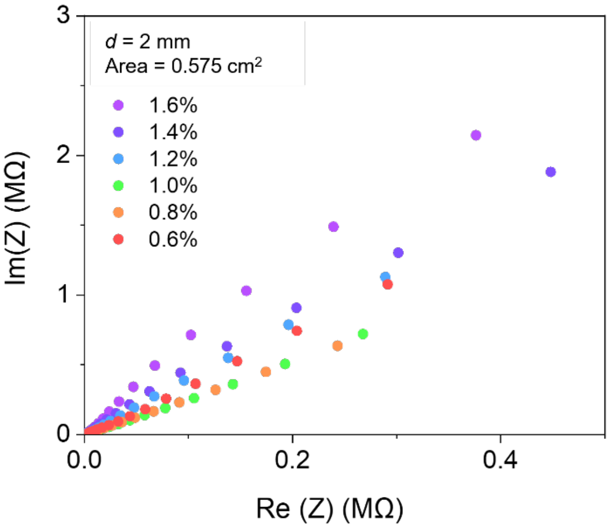

Figure S2. Nyquist plot of ι-CGN electrolyte with different concentrations of ι-CGN (0.6-1.6%) before crosslinking.

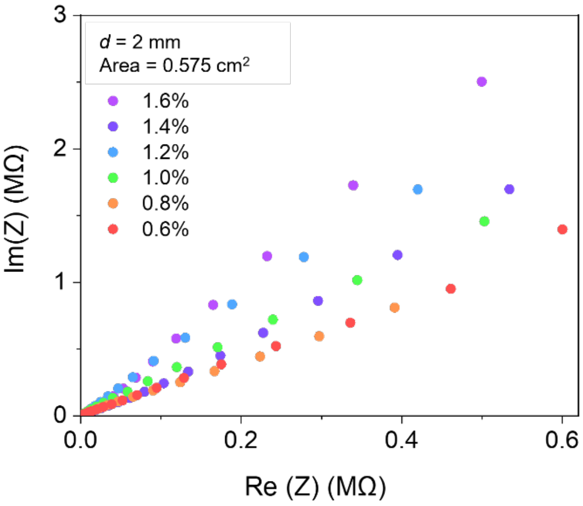

Figure S3. Nyquist plot of ι-CGN electrolyte with different concentrations of ι-CGN (0.6-1.6%) after crosslinking.

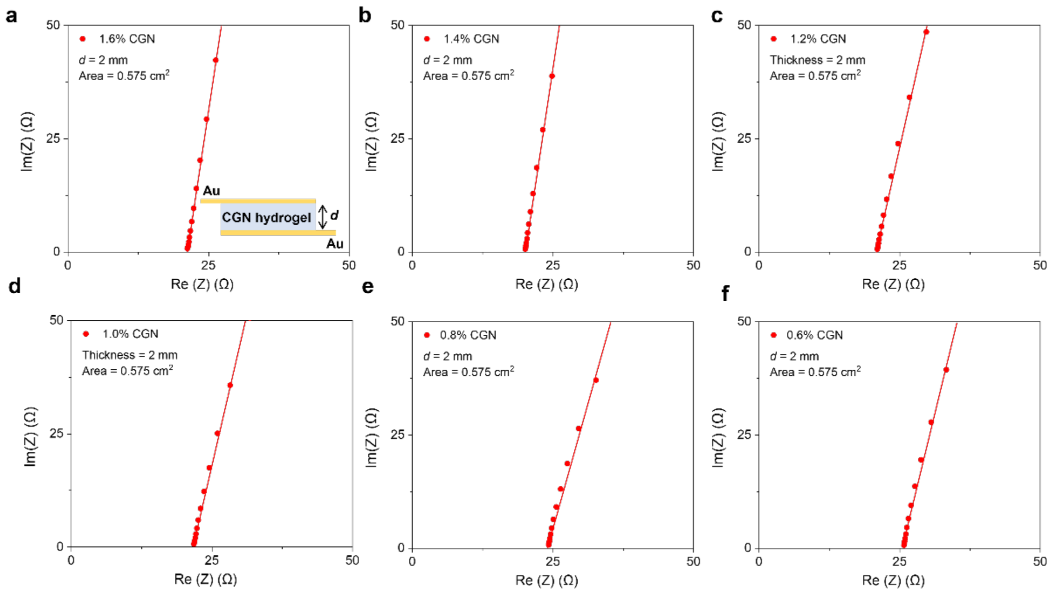

Figure S4. Zoomed-in Nyquist plot of ι-CGN electrolyte with different concentrations of ι-CGN (0.6-1.6%) before crosslinking, as reported in Fig. S2.

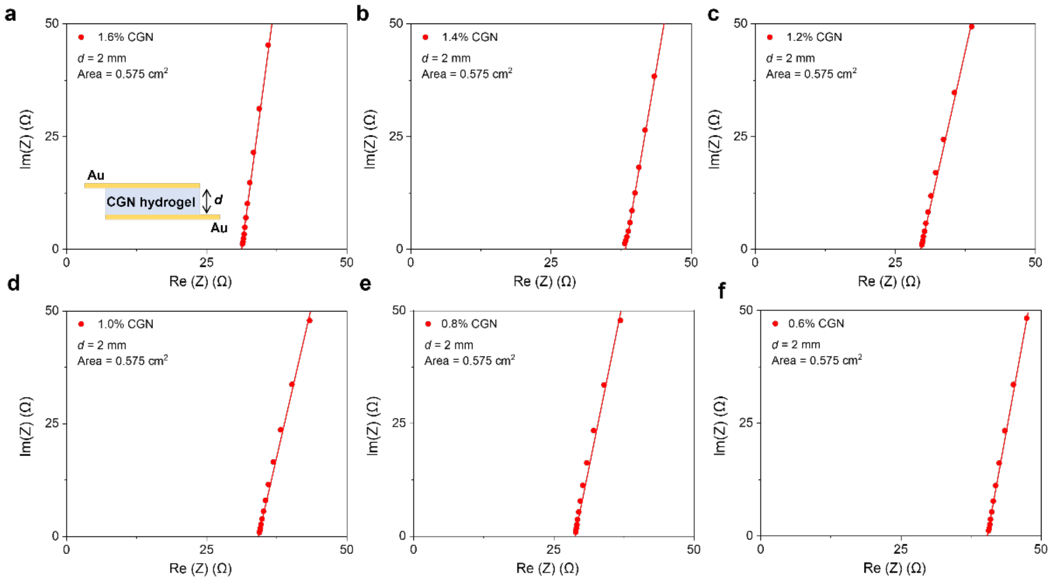

Figure S5. Zoomed-in Nyquist plot of ι-CGN electrolyte with different concentrations of ι-CGN (0.6-1.6%) after cross-linking zooming up by Figure S3.

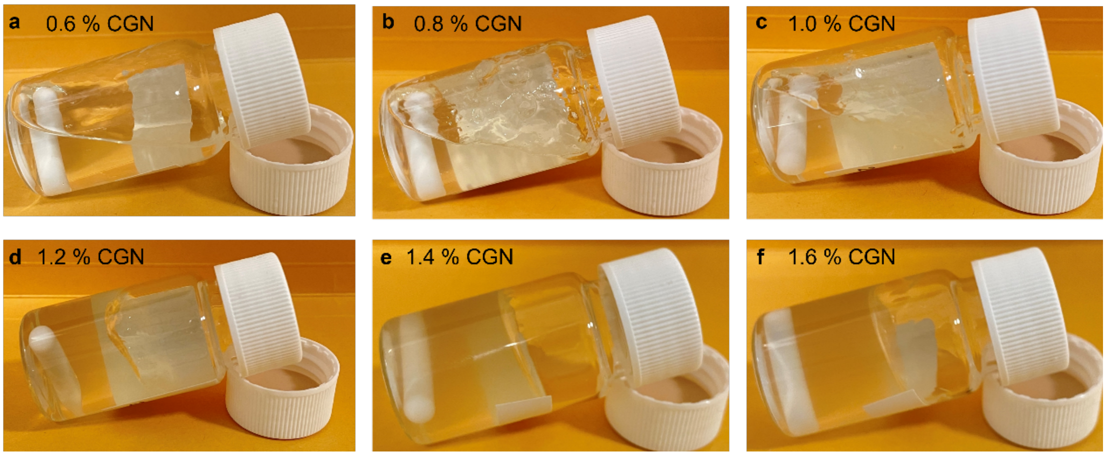

Figure S6. Photographs of the ι-CGN electrolyte at different concentrations of ι-CGN (0.6-1.6%) before crosslinking. Electrolytes with lower ι-CGN concentrations (0.6-1.0 wt.%) exhibit low viscosity and behave like fluid gels, whereas those with higher concentrations (1.2-1.6 wt.%) demonstrate higher viscosity and non-flowing properties at room temperature.

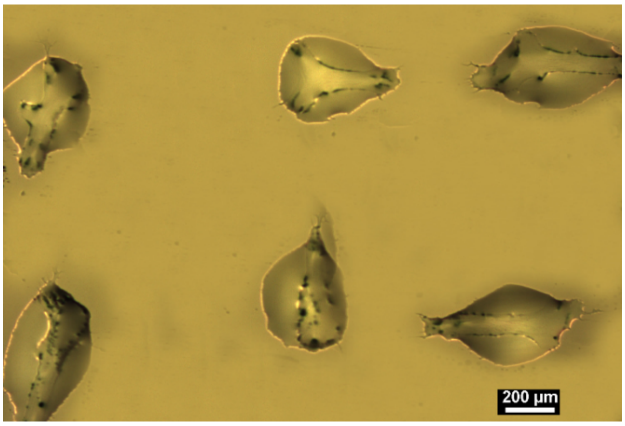

Figure S7. Photograph of the printed ι-CGN electrolyte. The ι-CGN electrolyte with 1.2 wt.% can be printed into 200-300 µm round shapes.

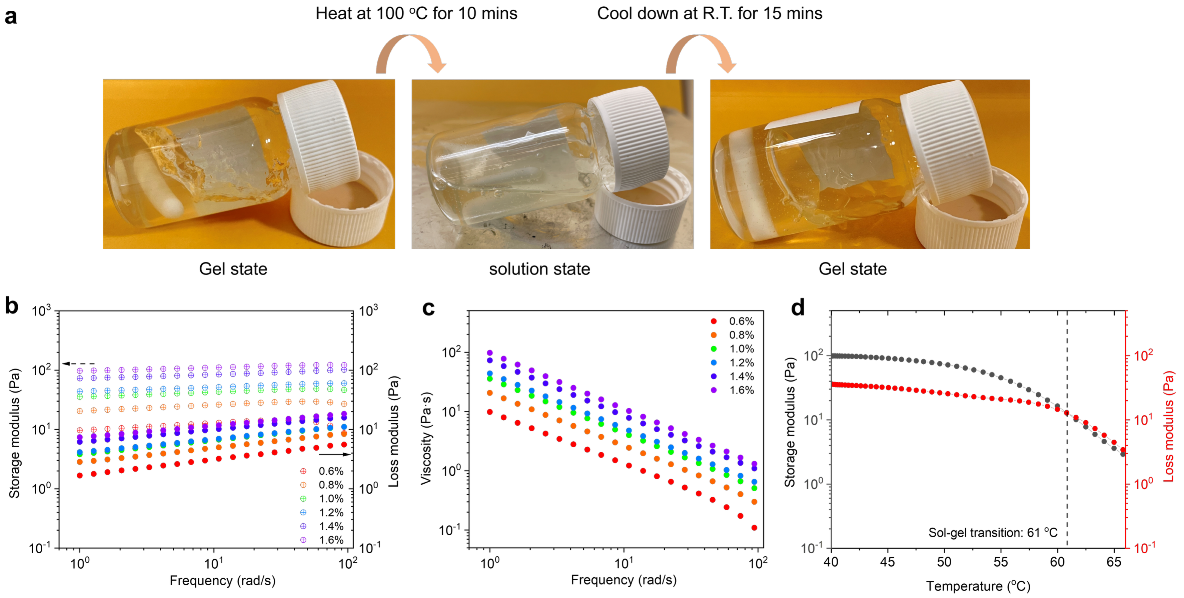

Figure S8. a) Photograph illustrating the gel-to-liquid-to-gel transition of the ι-CGN electrolyte with temperature. The electrolyte transitions to a solution when heated to 100 °C for 10 minutes and reverts to a gel upon cooling to room temperature. b) Storage and loss moduli as a function of frequency for the *ι*-CGN electrolyte before cross-linking at different concentrations of *ι*-CGN. c) Complex viscosity as a function of frequency for the *ι*-CGN electrolyte before cross-linking at different concentrations of *ι*-CGN. d) Storage and loss moduli as a function of temperature for the *ι*-CGN electrolyte.

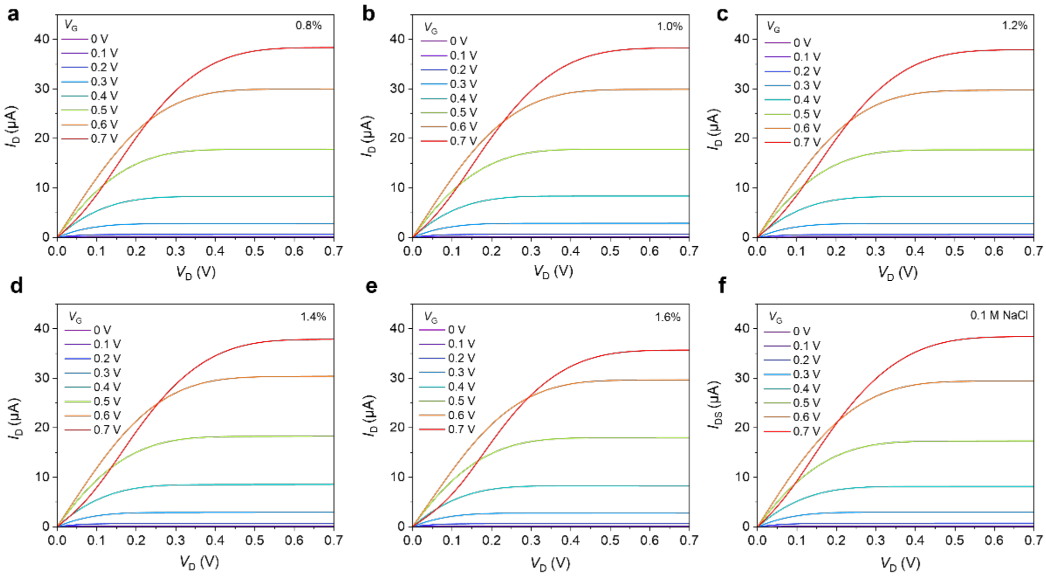

Figure S9. a-e) Output curves of BBL-based OECTs with ι-CGN solid-state electrolyte with different concentrations of ι-CGN (0.8-1.6%) and f) with 0.1 M NaCl aqueous electrolyte.

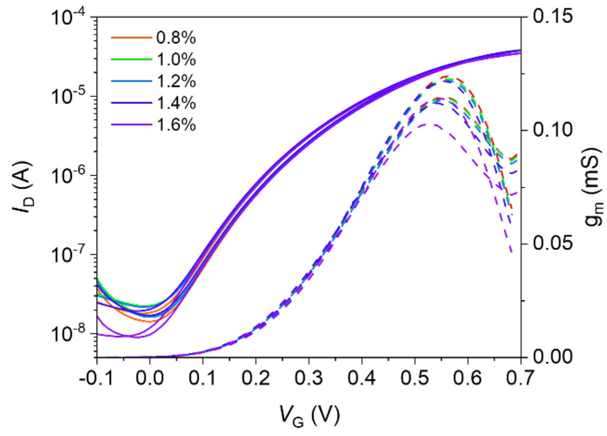

Figure S10. Transfer characteristics and transconductance of BBL OECTs with ι-CGN electrolyte with different concentrations of ι-CGN (0.8-1.6%) at V_{DS} = -0.7 V.

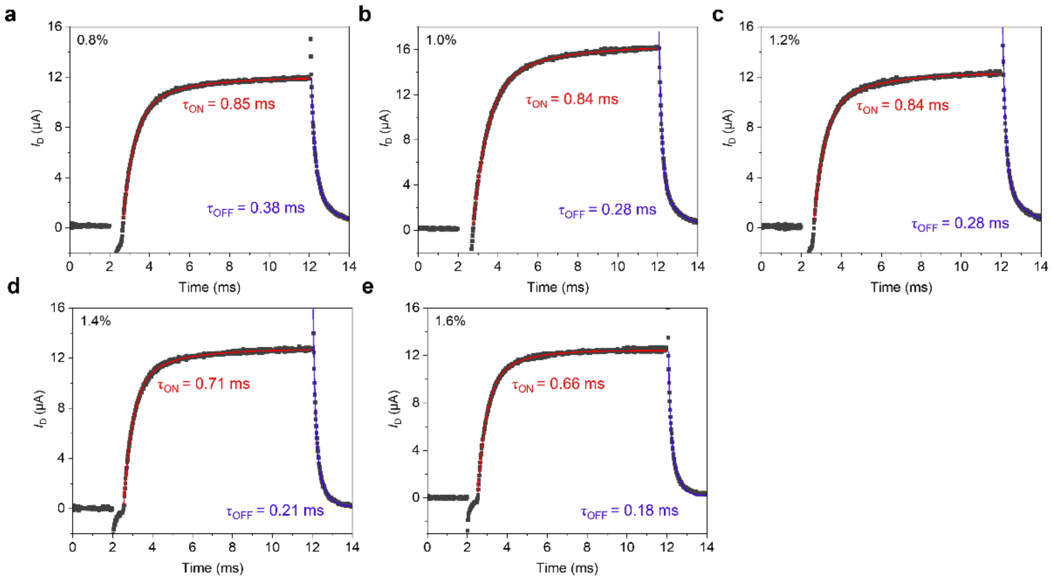

Figure S11. Transient response of BBL OECTs with ι-CGN solid-state electrolyte with different concentrations of ι-CGN (0.8-1.6%).

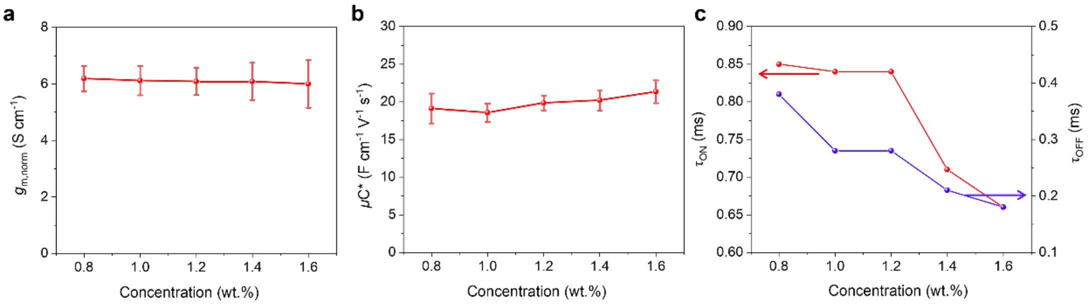

Figure S12. a) Normalized transconductance ($g_{m,norm}$) of BBL-based OECTs with ι-CGN solid-state electrolyte as a function of ι-CGN concentration. b) Product of mobility and volumetric capacitance (μC^*) of BBL-based OECTs with ι-CGN solid-state electrolyte as a function of ι-CGN concentration. (c) Transient response of BBL OECTs with ι-CGN solid-state electrolyte as a function of concentration. All OECTs have the same channel geometry (W = 100 μm, L = 10 μm, and d = 20 nm). Error bars represent the standard deviation from ten experimental replicates.

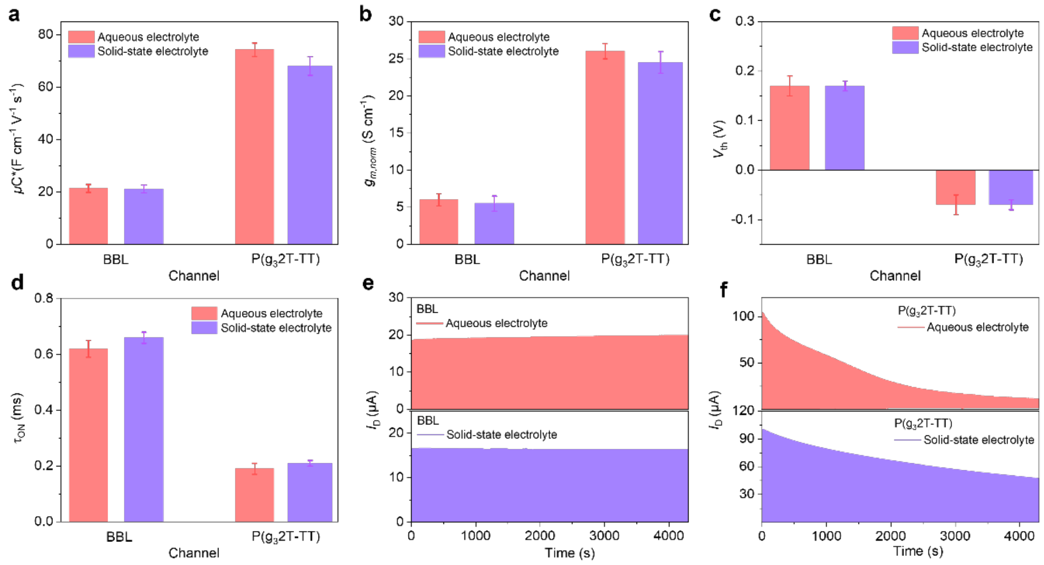

Figure S13. Comparison of OECT performance with ι-CGN solid-state electrolyte and 0.1 M NaCl solution electrolyte. a) μC*, b) $g_{m,norm}$, c) V_{th}, and d) τ_{ON} of BBL OECTs and P(g$_3$2T-TT) OECTs with ι-CGN solid-state and NaCl solution electrolytes. Stability test of OECTs with gate voltage pulses (0.6 V, 6.48 s length) applied for over 70 minutes: e) BBL OECTs and f) P(g$_3$2T-TT) OECTs. The n-type OECTs have a channel geometry of W/L = 100 μm/10 μm and a thickness of 20 nm, while the p-type OECTs have a geometry of W/L = 100 μm/10 μm and a thickness of 20 nm. Error bars represent the standard deviation from ten experimental replicates.

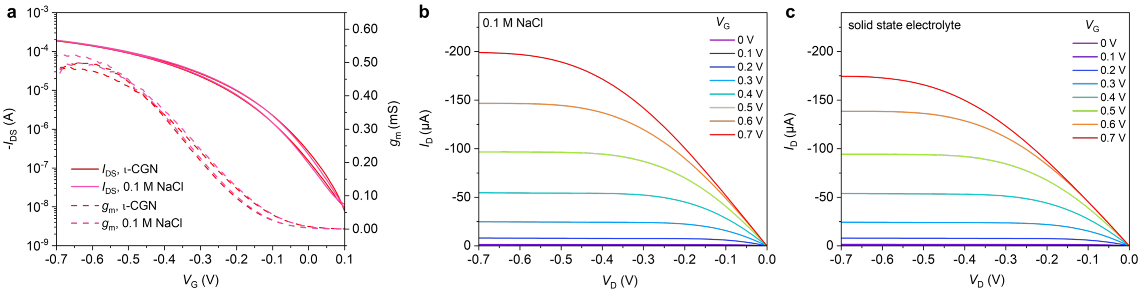

Figure S14. a) Transfer characteristics and transconductance of P(g$_3$2T-TT) OECTs with 0.1 M NaCl and ι-CGN electrolyte at V_{DS} = -0.7 V. Output curves of P(g$_3$2T-TT)-based OECTs with b) 0.1 M NaCl and c) ι-CGN solid-state electrolyte.

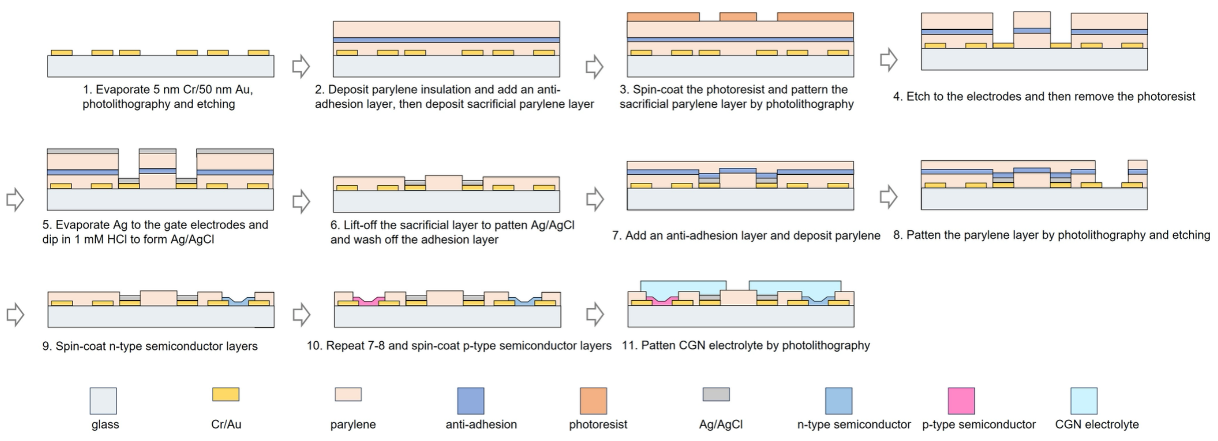

Figure S15. Fabrication schematic of the compact solid-state circuits (inverter, NAND, NOR, and half-adder) based on ι-CGN solid-state electrolyte.

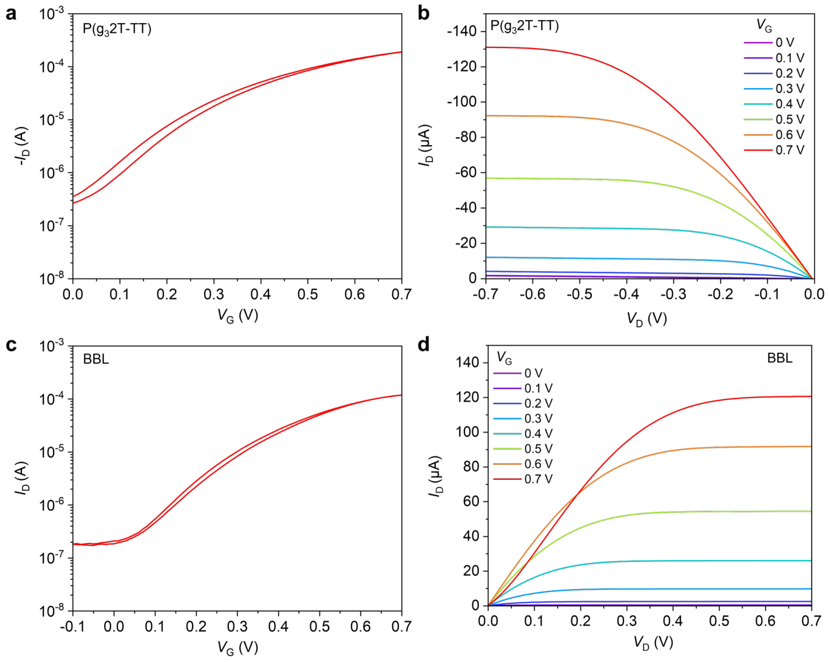

Figure S16. Output and transfer characteristics of a,b) P(g$_3$2T-TT)-based OECTs (W = 50 μm, L = 4 μm, and d = 5 nm) and c,d) BBL-based OECTs (W = 200 μm, L = 4 μm, and d = 20 nm).

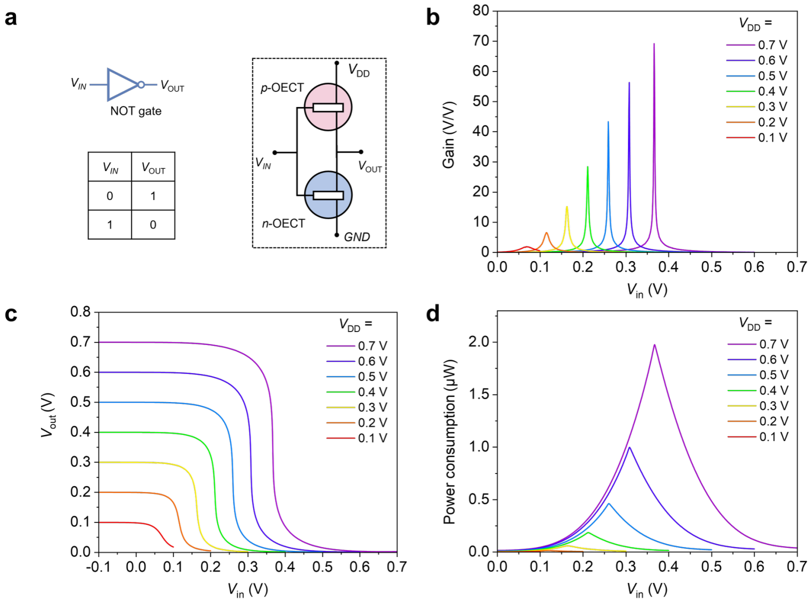

Figure S17. Performance of OECT-based complementary inverters with ι-CGN electrolyte. a) Schematic of the NOT gate (inverter). b) Voltage gains of the inverter at different supply voltages. c) Typical voltage transfer characteristics of the printed inverter at different supply voltages (0.1-0.7 V). d) Power consumption of the inverter at different supply voltages.

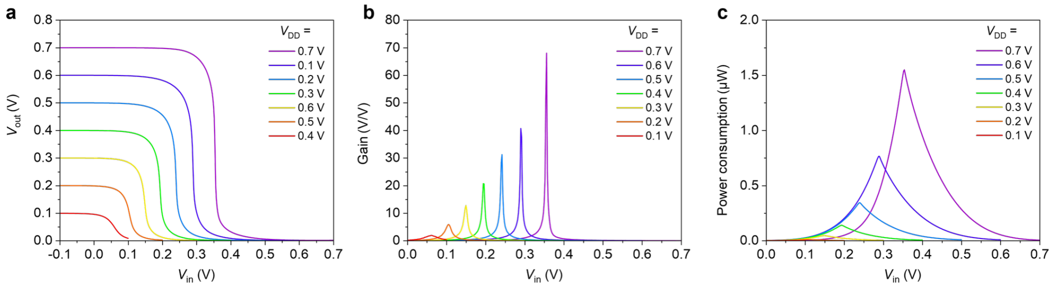

Figure S18. Performance of OECT-based complementary inverters with 0.1 M NaCl electrolyte. a) Typical voltage transfer characteristics (VTC) of the printed inverter at different supply voltages (0.1-0.7 V). b) Voltage gains of the inverter at different supply voltages. c) Power consumption of the inverter at different supply voltages.

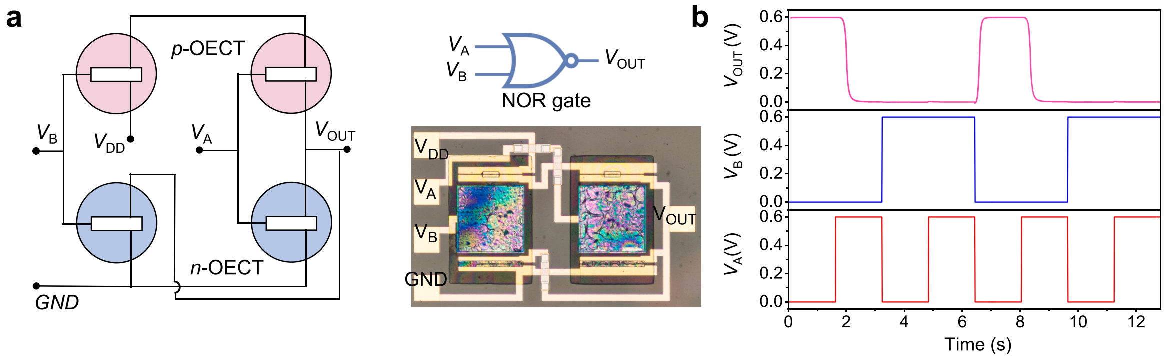

Figure S19. a) Schematic and photograph of a NOR gate based on 4 OECTs. The compact device (500 × 800 μm^2) comprises two p-type P(g$_3$2T-TT) OECTs and two n-type BBL OECTs with a patterned solid-state ι-CGN electrolyte. The gate electrode is shared by the p- and n-type channels. b) Voltage output characteristics of the NOR gate.

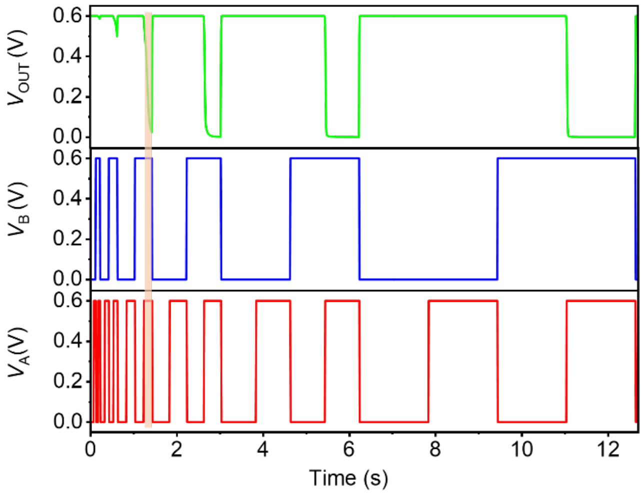

Figure S20. Screening of NAND gate operation speed. The correct logic functions are achieved within a time scale of 0.4 s.

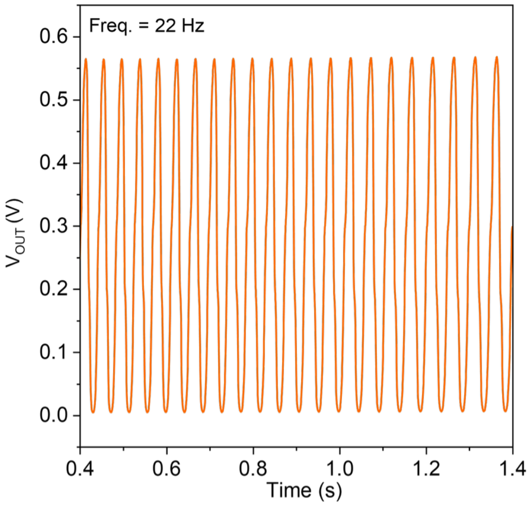

Figure S21. Output voltage of an encapsulated solid-state OECN recorded at I_{in} = 1 μA and V_{DD} = 0.6 V, and having OECT geometry as follows: P(g$_3$2T-TT) OECTs with W = 50 μm, L = 4 μm, and d = 5 nm, and BBL OECTs with W = 200 μm, L = 4 μm, and d = 20 nm.

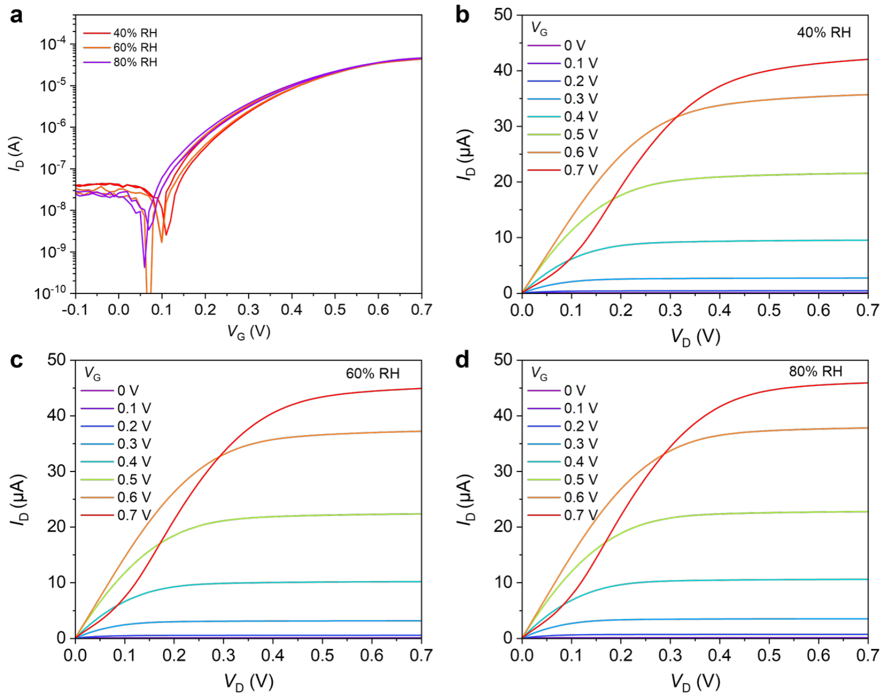

Figure S22. a) Transfer characteristics and transconductance of BBL-based OECTs with solid-state ι-CGN electrolyte at V_{DS} = -0.7 V under different relative humidity (RH) conditions. b-d) Output curves of BBL-based OECTs with solid-state ι-CGN electrolyte at 40% RH, 60% RH, and 80% RH. The transfer and output curves of the BBL OECTs remained nearly identical across the different humidity conditions. These results demonstrate the robustness of our solid-state electrolyte under varying humidity levels.

Table S1. Comparison of OECT performance with solid-state electrolyte and NaCl aqueous electrolyte.

**Active materials**	**Electrolyte**	**W/L (μm/μm)**	***d* (nm)**	**$g_{m,norm}$ (S/cm)**	**τ (ms)**	**Ref.**
P3HT	PVDF/EMIMTFSI	1000/100	110	42	950	[1]
	PEO/P_{14}:TFSI	2000/10	15	4.13	-	[2]
	KCl	1000/10	200	0.24	-	[3]
P(g2T-TT)	PEO/P_{14}:TFSI	2000/10	15	0.48	3	[2]
	NaCl	100/10	340	8.5	0.42	[4]
P($g_4$2T-T)	PQ-10	2000/200	20	8.5	48	[5]
	NaCl	100/10	20	80	0.074	[6]
P3CPT	PVDF-co-HFP/EMIMTFSI	106/12	35	20.9	4.31	[7]
	PVDF-HFP/ [EMIM][TFSI]	111/11	35	20.6	2.59	[7]
	Gelatin/[MTEOA][$MeOSO_3$]	109/12	35	17.9	0.47	[7]
	NaCl	39000/20	30	2.27	-	[8]
	NaCl	108/23	35	7.06	0.11	[7]
p(g1T2-g5T2)	PVDF-HFP/ [EMIM][TFSI]	100/10	28	19	0.34	[9]
	Gelatin/[MTEOA][$MeOSO_3$]	107/12	15	36.5	0.36	[7]
	NaCl	100/10	65	157	-	[10]
	NaCl	70/8	15	21.6	0.22	[7]
pgBTTT	PVDF-co-HFP/EMIMTFSI	118/15	47	100	6.42	[7]
	PVDF-HFP/ [EMIM][TFSI]	99/13	47	193	2.15	[7]
	Gelatin/[MTEOA][$MeOSO_3$]	97/12	47	202	3.97	[7]
	NaCl	100/10	108	185	-	[11]
	NaCl	116/15	47	144	0.43	[7]
PEDOT:PSS	PVDF-co-HFP/EMIMTFSI	1000/200	80	70	3.87	[1]
	PVA/NaCl	1000/200	2000	54	6.8	[12]
	NaCl	100/10	640	2.6	0.1	[13,14]
PEDOT:PSS/EG/ GOPS	Gelatin/[MTEOA][$MeOSO_3$]	106/12	53	122	0.40	[7]
	NaCl	102/12	53	132	0.15	[7]
BBL	PSSNa	2000/200	250	0.72	145	[5]
	Gelatin/[MTEOA][$MeOSO_3$]	110/12	36	0.7	0.54	[7]
	NaCl	20/10	20	11.1	0.38	[6]
**P($g_3$2T-TT)**	**ι-CGN electrolyte**	**100/10**	**20**	**24.5**	**0.21**	**This work**
	**NaCl**	**100/10**	**20**	**26**	**0.19**	**This work**
**BBL**	**ι-CGN electrolyte**	**100/10**	**20**	**5.5**	**0.66**	**This work**
	**NaCl**	**100/10**	**20**	**6.0**	**0.62**	**This work**

Table S2. Comparison of solid-state electrolyte performance in terms of ionic conductivity and pattern size.

Solid electrolytes	Ionic conductivity (mS/cm)	Minimum line width (μm)	Size of electrolyte (μm^2)	Device application	τ_{ON} (ms)	Fabrication method	Ref.
PEGDA/EMIMTFSI	-	2000	2000 × 2000	OECTs	27	UV exposure with mask	[15]
PEGDA/EMIMTFSI	2.62	800	800 × 800	Solid-state on-chip microsupercap	-	Jet printing	[16]
Chitosan	-	50	160 × 50	Ion-gated organic transistors (IGTs)	46	Photo-lithography with photoresist and plasma etching	[17]
PDMAPS/PAA/ILs	-	5000	5000 × 10000	Stretchable OECTs	-	Printing	[18]
PS-PEO-PS/EMIMTFSI	-	800	800 × 2500	OTFTs	-	Aerosol jet printing	[19]
PEO/LiTFSI	-	-	-	FETs	-	Spin-coating	[20]
Chitosan/Dextran:$LiClO_4$	0.56	4000	4000 × 6000	OECTs	-	Drop-casting	[21]
Glycerol gel	1.5	500	500 × 1000	OECTs	-	UV exposure with mask	[22]
PSSNa	16	1000	1000 × 2000	OECTs	145	Screen-printing	[5]
PQ-10	8.6	1000	1000 × 2000	OECTs	48	Screen-printing	[5]
PNIPAm/EMIMEtSO_4	24	100	200 × 300	OECTs	-	Direct photo-lithography	[23]
Gelatin/Glycerol	5×10^{-3}	-	-	OECTs	1.2	Drop-casting	[24]
Gelatin/[MTEOA][MeOSO_3]	0.56	-	-	OECTs	0.2	Drop-casting	[7]
PVDF-co-HFP/EMIM TFSI	0.7	-	-	OECTs	1570	Drop-casting	[25]
***ι*-CGN solid-state electrolyte**	12	**15**	**30 × 30**	**OECTs**	**0.19**	**Direct photo-lithography**	**This work**

Table S3. Comparison of the electrical performance of BBL OECTs and P(g$_3$2T-TT) OECTs using ι-CGN solid-state electrolyte and 0.1 M NaCl aqueous electrolyte.

Polymer	Electrolyte	μC^* (F cm^{-1} V^{-1} s^{-1})	$g_{m,norm}$ (S cm^{-1})	V_{th} (V)	$\tau_{ON,90\%}$ (ms)	$\tau_{OFF,90\%}$ (ms)	τ_{ON} (ms)	τ_{OFF} (ms)
P(g$_3$2T-TT)	NaCl	74.29	26	-0.07	0.52	0.074	0.19	0.036
	CGN	68.06	24.5	-0.08	0.56	0.084	0.21	0.038
BBL	NaCl	21.43	6.0	0.17	2.25	0.87	0.62	0.29
	CGN	21.15	5.5	0.17	2.28	0.49	0.66	0.18

Table S4. Comparison of transient times (τ_{ON}, τ_{OFF}, $\tau_{ON,90\%}$, and $\tau_{OFF,90\%}$) of BBL OECTs with ι-CGN solid-state electrolyte at different carrageenan concentrations (0.8-1.6%).

Concentration	$\tau_{ON,90\%}$	τ_{ON}	$\tau_{OFF,90\%}$	τ_{OFF}
0.8%	2.98	0.85	1.05	0.38
1.0%	3.50	0.84	0.74	0.28
1.2%	3.06	0.84	0.76	0.28
1.4%	2.52	0.71	0.47	0.21
1.6%	2.28	0.66	0.49	0.18

Table S5. Statistics of typical complementary circuits, including the number of OECTs and the inverter size.

Device	Electrolyte	OECT numbers	Inverter size[a] (μm^2)	Ref.
Ring oscillator	0.1 M KCl (aqueous)	10	800 × 600	[26]
NAND/NOR	0.1 M NaCl (aqueous)	4	-	[27]
Amplifier array	PBS (aqueous)	16	60 × 60	[28]
Inverter	0.1 M NaCl (aqueous)	2	30 × 30	[29]
NAND/NOR	PEGDA:EMIMTFSI (solid)	4	300 × 300	[30]
Inverter	PQ-10/PSSNa (solid)	2	20000 × 10000	[5]
**NAND/NOR/Half adder**	***ι*-CGN electrolyte (solid)**	**18**	**250 × 400**	**This work**

[a]The gate area can't be calculated into inverter size when the electrolyte is aqueous with the floating gate.

Table S6. Comparison of complementary logic circuits based on OECTs.

Type of OECT	NAND gate speed (s)	Half adder speed (s)	Ref.
Vertical OECT	0.05	-	[26]
Vertical OECT	< 0.05	-	[30]
Planar OECT (hydrogel polymer, aqueous electrolyte)	0.5	-	[27]
**Planar OECT (solid-state electrolyte)**	**0.4**	**1.6**	**This work**

Table S7. The truth table of the NAND gate, the NOR gate, the 4-input NAND gate, and the half adder.

V_A	0	0	1	1
V_B	0	1	0	1
V_{out} (NAND)	1	1	1	0
V_{out} (NOR)	1	0	0	0

V_A	0	1	0	1
V_B	0	0	1	1
Sum	0	1	1	0
Carry	0	0	0	1

4-input NAND

V_A	1	0	1	0	1	0	1	0	1	0	1	0	1	0	1	0
V_B	1	1	0	0	1	1	0	0	1	1	0	0	1	1	0	0
V_C	1	1	1	1	0	0	0	0	1	1	1	1	0	0	0	0
V_D	1	1	1	1	1	1	1	1	0	0	0	0	0	0	0	0
V_{out}	0	1	1	1	1	1	1	1	1	1	1	1	1	1	1	1

References

[1] S. Chen, A. Surendran, X. Wu, W. L. Leong, *Adv. Funct. Mater.* **2020**, *30*, 2006186.

[2] Q. Thiburce, A. Giovannitti, I. McCulloch, A. J. Campbell, *Nano Lett.* **2019**, *19*, 1712.

[3] J. Wagner, Y. Song, T. Lee, H. E. Katz, *Electrochem. Sci. Adv.* **2022**, *2*, e2100165.

[4] A. Giovannitti, D.-T. Sbircea, S. Inal, C. B. Nielsen, E. Bandiello, D. A. Hanifi, M. Sessolo, G. G. Malliaras, I. McCulloch, J. Rivnay, *Proc. Natl. Acad. Sci.* **2016**, *113*, 12017.

[5] C.-Y. Yang, D. Tu, T.-P. Ruoko, J. Y. Gerasimov, H.-Y. Wu, P. C. Harikesh, M. Massetti, M.-A. Stoeckel, R. Kroon, C. Müller, M. Berggren, S. Fabiano, *Adv. Electron. Mater.* **2022**, *8*, 2100907.

[6] H.-Y. Wu, C.-Y. Yang, Q. Li, N. B. Kolhe, X. Strakosas, M.-A. Stoeckel, Z. Wu, W. Jin, M. Savvakis, R. Kroon, D. Tu, H. Y. Woo, M. Berggren, S. A. Jenekhe, S. Fabiano, *Adv. Mater.* **2022**, *34*, 2106235.

[7] C. G. Tang, R. Wu, Y. Chen, Z. Zhou, Q. He, T. Li, X. Wu, K. Hou, C. J. Kousseff, I. McCulloch, W. L. Leong, *Adv. Mater.* **2024**, *36*, 2405556.

[8] H. Sun, M. Vagin, S. Wang, X. Crispin, R. Forchheimer, M. Berggren, S. Fabiano, *Adv. Mater.* **2018**, *30*, 1704916.

[9] X. Wu, S. Chen, M. Moser, A. Moudgil, S. Griggs, A. Marks, T. Li, I. McCulloch, W. L. Leong, *Adv. Funct. Mater.* **2023**, *33*, 2209354.

[10] M. Moser, T. C. Hidalgo, J. Surgailis, J. Gladisch, S. Ghosh, R. Sheelamanthula, Q. Thiburce, A. Giovannitti, A. Salleo, N. Gasparini, A. Wadsworth, I. Zozoulenko, M. Berggren, E. Stavrinidou, S. Inal, I. McCulloch, *Adv. Mater.* **2020**, *32*, 2002748.

[11] R. K. Hallani, B. D. Paulsen, A. J. I. Petty, R. Sheelamanthula, M. Moser, K. J. Thorley, W. Sohn, R. B. Rashid, A. Savva, S. Moro, J. P. Parker, O. Drury, M. Alsufyani, M. Neophytou, J. Kosco, S. Inal, G. Costantini, J. Rivnay, I. McCulloch, *J. Am. Chem. Soc.* **2021**, *143*, 11007.

[12] J. Ko, X. Wu, A. Surendran, B. T. Muhammad, W. L. Leong, *ACS Appl. Mater. Interfaces* **2020**, *12*, 33979.

[13] J. Rivnay, P. Leleux, M. Sessolo, D. Khodagholy, T. Hervé, M. Fiocchi, G. G. Malliaras, *Adv. Mater.* **2013**, *25*, 7010.

[14] D. Khodagholy, M. Gurfinkel, E. Stavrinidou, P. Leleux, T. Herve, S. Sanaur, G. G. Malliaras, *Appl. Phys. Lett.* **2011**, *99*, 163304.

[15] S. Y. Jeong, J. W. Moon, S. Lee, Z. Wu, S. H. Park, J. H. Cho, H. Y. Woo, *Adv. Electron. Mater.* **2023**, *9*, 2300053.

[16] J. Kang, Y.-W. Jang, S. H. Moon, Y. Kang, J. Kim, Y.-H. Kim, S. K. Park, *Adv. Sci.* **2022**, *9*, 2103275.

[17] G. D. Spyropoulos, J. N. Gelinas, D. Khodagholy, *Sci. Adv.* **2019**, *5*, eaau7378.

[18] D. Liu, X. Tian, J. Bai, S. Wang, S. Dai, Y. Wang, Z. Wang, S. Zhang, *Nat. Electron.* **2024**, *7*, 1176.

[19] J. H. Cho, J. Lee, Y. Xia, B. Kim, Y. He, M. J. Renn, T. P. Lodge, C. Daniel Frisbie, *Nat. Mater.* **2008**, *7*, 900.

[20] M. J. Panzer, C. D. Frisbie, *J. Am. Chem. Soc.* **2007**, *129*, 6599.

[21] B. Sun, S. F. Wan Muhamad Hatta, N. Soin, M. F. Z. B. A. Kadir, F. A. Md Rezali, S. N. Aidit, L. Y. Ma, Q. Ma, *ACS Appl. Electron. Mater.* **2024**, *6*, 2336.

[22] H. Lee, S. Lee, W. Lee, T. Yokota, K. Fukuda, T. Someya, *Adv. Funct. Mater.* **2019**, *29*, 1906982.

[23] A. Weissbach, L. M. Bongartz, M. Cucchi, H. Tseng, K. Leo, H. Kleemann, *J. Mater. Chem. C* **2022**, *10*, 2656.

[24] T. Nguyen-Dang, K. Harrison, A. Lill, A. Dixon, E. Lewis, J. Vollbrecht, T. Hachisu, S. Biswas, Y. Visell, T.-Q. Nguyen, *Adv. Electron. Mater.* **2021**, *7*, 2100519.

[25] M. Azimi, C. Kim, J. Fan, F. Cicoira, *Faraday Discuss.* **2023**, *246*, 540.

[26] W. Huang, J. Chen, Y. Yao, D. Zheng, X. Ji, L.-W. Feng, D. Moore, N. R. Glavin, M. Xie, Y. Chen, R. M. Pankow, A. Surendran, Z. Wang, Y. Xia, L. Bai, J. Rivnay, J. Ping, X. Guo, Y. Cheng, T. J. Marks, A. Facchetti, *Nature* **2023**, *613*, 496.

[27] P. Li, W. Sun, J. Li, J.-P. Chen, X. Wang, Z. Mei, G. Jin, Y. Lei, R. Xin, M. Yang, J. Xu, X. Pan, C. Song, X.-Y. Deng, X. Lei, K. Liu, X. Wang, Y. Zheng, J. Zhu, S. Lv, Z. Zhang, X. Dai, T. Lei, *Science* **2024**, *384*, 557.

[28] I. Uguz, D. Ohayon, S. Yilmaz, S. Griggs, R. Sheelamanthula, J. D. Fabbri, I. McCulloch, S. Inal, K. L. Shepard, *Sci. Adv.* **2024**, *10*, eadi9710.

[29] R. B. Rashid, W. Du, S. Griggs, I. P. Maria, I. McCulloch, J. Rivnay, *Sci. Adv.* **2021**, *7*, eabh1055.

[30] J. Kim, R. M. Pankow, Y. Cho, I. D. Duplessis, F. Qin, D. Meli, R. Daso, D. Zheng, W. Huang, J. Rivnay, T. J. Marks, A. Facchetti, *Nat. Electron.* **2024**, *7*, 234.
